# Hollow Nanocages of Ni_*x*_Co_1−*x*_Se for Efficient Zinc–Air Batteries and Overall Water Splitting

**DOI:** 10.1007/s40820-019-0258-0

**Published:** 2019-03-27

**Authors:** Zhengxin Qian, Yinghuan Chen, Zhenghua Tang, Zhen Liu, Xiufang Wang, Yong Tian, Wei Gao

**Affiliations:** 1 School of Pharmacy, Guangdong Pharmaceutical University, Guangzhou, 510006 Guangdong People’s Republic of China; 2 Guangzhou Key Laboratory for Surface Chemistry of Energy Materials, New Energy Research Institute, School of Environment and Energy, South China University of Technology, Guangzhou Higher Education Mega Center, Guangzhou, 510006 Guangdong People’s Republic of China; 3 Guangdong Engineering and Technology Research Center for Surface Chemistry of Energy Materials, School of Environment and Energy, South China University of Technology, Guangzhou Higher Education Mega Centre, Guangzhou, 510006 Guangdong People’s Republic of China; 4 Department of Physics and Engineering, Frostburg State University, Frostburg, MD 21532-2303 USA

**Keywords:** Ni_*x*_Co_1−*x*_Se hollow nanocages, Oxygen evolution reaction, Hydrogen evolution reaction, Rechargeable/all-solid-state zinc–air battery, Overall water splitting

## Abstract

Developing Earth-abundant, highly efficient, and anti-corrosion electrocatalysts to boost the oxygen evolution reaction (OER), oxygen reduction reaction (ORR), and hydrogen evolution reaction (HER) for the Zn–air battery (ZAB) and for overall water splitting is imperative. In this study, a novel process starting with Cu_2_O cubes was developed to fabricate hollow Ni_*x*_Co_1−*x*_Se nanocages as trifunctional electrocatalysts for the OER, ORR, and HER and a reasonable formation mechanism was proposed. The Ni_0.2_Co_0.8_Se nanocages exhibited higher OER activity than its counterparts with the low overpotential of 280 mV at 10 mA cm^−2^. It also outperformed the other samples in the HER test with a low overpotential of 73 mV at 10 mA cm^−2^. As an air–cathode of a self-assembled rechargeable ZAB, it exhibited good performance, such as an ultralong cycling lifetime of > 50 h, a high round-trip efficiency of 60.86%, and a high power density of 223.5 mW cm^−2^. For the application in self-made all-solid-state ZAB, it also demonstrated excellent performance with a power density of 41.03 mW cm^−2^ and an open-circuit voltage of 1.428 V. In addition, Ni_0.2_Co_0.8_Se nanocages had superior performance in a practical overall water splitting, in which only 1.592 V was needed to achieve a current density of 10 mA cm^−2^. These results show that hollow Ni_*x*_Co_1−*x*_Se nanocages with an optimized Ni-to-Co ratio are a promising cost-effective and high-efficiency electrocatalyst for ZABs and overall water splitting in alkaline solutions.

**Electronic supplementary material:**

The online version of this article (10.1007/s40820-019-0258-0) contains supplementary material, which is available to authorized users.

## Highlights

- A facile strategy for fabricating Ni_*x*_Co_1−*x*_Se hollow nanocages was developed, and the formation mechanism was well explained.
- Ni_0.2_Co_0.8_Se outperformed a Pt/C + RuO_2_ catalyst in rechargeable and all-solid-state Zn–air battery tests, as well as in overall water splitting.
- The hydrogen adsorption onto Ni_*x*_Co_1−*x*_Se was simulated, and Gibbs free energies were calculated.

**Electronic supplementary material:**

The online version of this article (10.1007/s40820-019-0258-0) contains supplementary material, which is available to authorized users.

## Graphical abstract

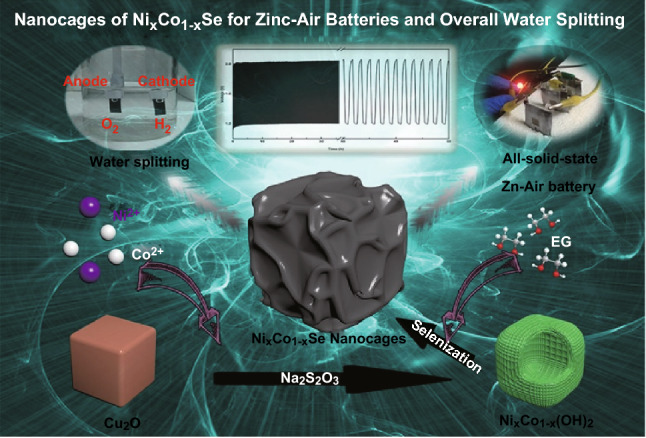

## Introduction

The rapid depletion and heavy reliance on fuel cells and associated global environmental concerns have motivated extensive research on the development of eco-friendly and sustainable energy technologies in the past decade. Among these technologies, Zn–air batteries (ZABs) and water-splitting devices have become viable eco-friendly energy technologies owing to recent advances in the preparation of highly active electrocatalysts [1–3]. The oxygen evolution reaction (OER) and oxygen reduction reaction (ORR) are the key reversible reactions occurring at the cathode of ZABs and largely determine the energy-conversion efficiency of ZABs [4]. The OER and the hydrogen evolution reaction (HER) are the two electrochemical reactions for catalyzing overall water splitting [5–7]. Pt-based materials have been widely considered as state-of-the-art electrocatalysts for the ORR and HER [8–12], while RuO_2_ and IrO_2_ are the standard high-efficiency OER catalysts [13, 14]. However, the large-scale commercial implementation of both Pt-based and Ru-/Ir-based materials has been significantly hampered by their scarcity, high cost, and poor long-term durability. Therefore, it is imperative to develop Earth-abundant, cost-effective, high-efficiency, and robust electrocatalysts [12, 15–24].

Among the various alternative materials, transition metal chalcogenides have been attracting increasing research attention, mainly owing to their high availability, low cost, and eco-friendliness [25–32]. In particular, because of the high conductivity of metallic Se compared with O and S, transition metal selenides (MSe, M = transition metal) have superior electrocatalytic performance to transition metal oxides and sulfides [30, 31]. Therefore, transition metal selenides have received tremendous research attentions from the electrocatalytic community. For instance, Zheng et al. developed a novel hot-injection process to precisely control the phase and composition of a series of Ni_*x*_Se nanocrystals and discovered that Ni_0.5_Se nanoparticles exhibited superior OER activity comparable to that of RuO_2_, that Ni_0.75_Se nanoparticles exhibited the best performance for the HER and ORR, and that both could be engineered for efficient rechargeable ZABs and water splitting [33]. Cao et al. demonstrated a facile strategy for in situ coupling of ultrafine Co_0.85_Se nanocrystals with N-doped C, and the as-prepared Co_0.85_Se@NC was employed as a trifunctional catalyst for the HER, ORR, and OER, exhibiting great potential for ZABs and water splitting [34]. Rather than using only one transition metal, recent studies showed that superior electrocatalytic performance for ZABs and water splitting could be achieved by employing mixed transition metal selenides. For example, Xu et al. [35] prepared a Ni–Fe diselenide (Ni_*x*_Fe_1−*x*_Se_2_) and used it as a templating precursor to form ultrathin nanosheets of the corresponding oxide, which exhibited a very low overpotential of only 195 mV in an alkaline solution at 10 mA cm^−2^ for the OER. Recently, Lv et al. [36] have designed Ni–Fe selenide (NiFeSe_2_) hollow nanoparticles, hollow nanochains [37], and Co–Fe selenide (CoFeSe_2_) nanosheets [38] for the OER, and the Co_0.4_Fe_0.6_Se nanosheets not only exhibited superior OER performance with a low overpotential of 217 mV at 10 mA cm^−2^ and a small Tafel slope of 41 mV dec^−1^ but also had a ultrahigh durability. There have been several reports of NiCoSe_2_-based materials for electrochemical energy storage and conversion. Yuan et al. reported monodisperse metallic NiCoSe_2_ hollow sub-microspheres for electrochemical supercapacitors [39]. NiCoSe_2−*x*_/N-doped C mushroom-like core/shell nanorods on N-doped C fiber were prepared by Li et al. [40] for overall water splitting, and a low cell voltage of 1.53 V to obtain a current density of 10 mA cm^−2^ was observed. Recently, Chen and Tan have directly grew ultrathin ternary selenide (CoNiSe_2_) nanorods on Ni foam, which delivered a current density of 100 mA cm^−2^ with an overpotential as low as 307 and 170 mV for the OER and HER, respectively, and eventually reduced the cell voltage in the full water-splitting reaction to 1.591 V to obtain a current density of 10 mA cm^−2^ [41]. Chen and Wang groups prepared a three-dimensional Ni–Co selenide (NiCoSe_2_) nanonetwork for the OER, and the overpotential at 10 mA cm^−2^ was 274 mV, exhibiting room for improvement [42].

Despite the progress regarding NiCoSe_2_, direct preparation of NiCoSe_2_ with precise manipulation of the morphology for ZABs and overall water splitting remains largely unexplored. Moreover, the stoichiometric ratio of Ni to Co has not been optimized for enhancing the synergistic catalytic effects. In light of the significant effects of the morphology, crystal structure, and stoichiometry on the electrocatalytic performance, a systematic investigation of NiCoSe_2_ with a well-defined surface structure and an optimized Ni/Co stoichiometry for establishing the structure–function relationship of NiCoSe_2_ materials is of great importance. This was the primary goal of the present study.

In this study, we employed a facile strategy to prepare a series of Ni_*x*_Co_1−*x*_Se samples with hollow cages and investigated them as trifunctional electrocatalysts for the OER, ORR, and HER. A novel process with Cu_2_O cubes as the starting material was developed to fabricate the Ni_x_Co_1-x_Se nanocages, and a reasonable formation mechanism was proposed. In electrochemical tests, Ni_0.2_Co_0.8_Se exhibited higher OER and HER activity than the other samples in the Ni_*x*_Co_1−*x*_Se series. To investigate the applications of the Ni_0.2_Co_0.8_Se sample, it was used as an air–cathode of a self-assembled rechargeable ZAB and an all-solid-state ZAB and employed as a catalyst for overall water splitting in an alkaline solution.

## Experimental Section

### Materials

Copper (II) chloride dihydrate (CuCl_2_·2H_2_O, 99%), sodium hydroxide (NaOH, ≥ 96.0%), *L*-ascorbic acid (AA, ≥ 99.7%), nickel (II) chloride hexahydrate (NiCl_2_·6H_2_O, ≥ 98.0%), cobalt (II) chloride hexahydrate (CoCl_2_·6H_2_O, ≥ 99.0%), polyvinylpyrrolidone (PVP, K30, 99%), anhydrous sodium thiosulfate (Na_2_S_2_O_3_, 99%), sodium selenite (Na_2_SeO_3_, ≥ 99.7%), absolute ethanol (≥ 99.7%), and ethylene glycol (EG, 99.0%) were used. Water was obtained from a Barnstead Nanopure water system (resistivity: 18.3 MΩ cm). All the chemicals were used as received, without further purification.

### Synthesis of Cu_2_O Cubes

Cu_2_O cubes were synthesized by following a previously reported procedure [43]. Typically, 341 mg of CuCl_2_·2H_2_O was first dissolved in 200 mL of Nanopure water. Then, the solution was heated to 55 °C and stirred for 30 min. Subsequently, 20 mL of a 2 M NaOH solution was slowly added to the aforementioned solution, forming a brown suspension. After 10 min of stirring, 20 mL of 0.6 M AA was added dropwise to the solution. The solution gradually changed from dark red to brick red, and the mixture was aged for 3 h. The formed precipitates were collected via suction filtration, washed with copious distilled water and ethanol 3–5 times, and eventually dried in vacuum at 35 °C overnight.

### Synthesis of Ni_0.2_Co_0.8_(OH)_2_ Nanocages

In a typical procedure, 100 mg of cuprous oxide was dissolved into a mixed solvent of absolute ethanol and Nanopure water (100 mL, volume ratio = 1:1) with 30 min of ultrasonic treatment. Then, 34 mg of NiCl_2_·6H_2_O and CoCl_2_·6H_2_O (molar ratio of 2:8) was added to the solution, with stirring. Subsequently, 3.33 g of PVP was dispersed in the resulting suspension under another 30 min of ultrasonic treatment. Then, 40 mL of 1 M Na_2_S_2_O_3_ was slowly added to the mixture. Upon the addition of an excessive amount of sodium thiosulfate solution, the mixture changed from orange–red to transparent green, indicating that cuprous oxide was converted into Ni_0.2_Co_0.8_(OH)_2_. The reaction was conducted for 10 min to ensure that it was complete. The product was then collected via centrifugation, washed with copious Nanopure water and ethanol 3–5 times, and eventually dried in a vacuum at 35 °C overnight. For the synthesis of Ni_0.5_Co_0.5_(OH)_2_, Ni_0.8_Co_0.2_(OH)_2_, Ni(OH)_2_, and Co(OH)_2_, the same procedure was adopted, but the molar ratio of Ni to Co was changed to 5:5, 8:2, 1:0, and 0:1, respectively.

### Synthesis of Ni_0.2_Co_0.8_Se Nanocages

In a typical procedure, 37 mg of Na_2_SeO_3_ was dissolved in a mixed solvent of Nanopure water and EG (10.0 mL, volume ratio = 1:1). Then, 10 mg of Ni_0.2_Co_0.8_(OH)_2_ was added to the solution, with 30 min of ultrasonication to ensure uniform dispersion. Subsequently, the mixture was transferred into an autoclave and kept at 200 °C for 6 h. Finally, after cooling to room temperature, the product was collected via centrifugation.

### Characterization

The morphologies and surface structures of the samples were observed via field emission scanning electron microscopy (SEM, Hitachi S-4800) and high-resolution transmission electron microscopy (HRTEM, Tecnai G2 F30). X-ray diffraction (XRD) patterns in the Bragg’s angle (2*θ*) range of 10°–90° were recorded using a Bruker D8 diffractometer with Cu K_α_ radiation (*λ* = 0.1541 nm). X-ray photoelectron spectroscopy (XPS) was conducted using an ESCALAB 250 photoelectron spectrometer (Thermo Fisher Scientific, USA).

### Electrochemistry

Electrochemical measurements were taken using a CHI 750E electrochemical workstation (CHI Instruments Inc.) in a 1 M KOH aqueous solution at ambient temperature. A three-electrode system was utilized in both HER and OER tests. Here, Ag/AgCl was used as the reference electrode [44–46], and C rod and C cloth electrodes were employed as the counter electrode and the working electrode, respectively. The catalyst ink was prepared as follows. Firstly, 10 mg of the catalyst was ultrasonically dispersed in 1000 μL of absolute ethanol, followed by the sequential addition of 900 μL of Nanopure water and 100 μL of Nafion (5%, Sigma-Aldrich), yielding a uniform suspension. Then, 20 μL of the suspension was cast dropwise onto a single-sided C cloth (1.5 × 0.5 cm^2^, load area of 0.5 cm^2^), followed by drying at room temperature. The catalyst loading was calculated as ~ 200 μg cm^−2^. The solution was saturated with N_2_ or O_2_ at least 30 min before each measurement. For the HER, the cyclic voltammetry (CV) test potential range was − 0.077 to 0.623 V (vs. reversible hydrogen electrode (RHE)), and the scan rate was 100 mV s^−1^. In addition, linear sweep voltammetry (LSV) was conducted in a potential range of − 0.477 to 0.323 V (vs. RHE), at a scan rate of 10 mV s^−1^. OER measurements were taken in the same manner as the HER measurements. LSV was performed in a N_2_-saturated 1 M KOH solution within the potential range of + 1.023 to + 2.023 V (vs. RHE), at a scan rate of 10 mV s^−1^. A relatively simple two-electrode system was used in the water-splitting test, where the same catalyst was loaded on two clip electrodes: an anode and a cathode. The operation method was similar to that for the OER test, but the LSV test voltage was 1.0–2.0 V. We recorded the chronoamperometric responses in a 1 M KOH solution for 40,000 s and performed an accelerated durability test (ADT), where the catalyst was cycled 1000 times in the potential range of + 1.023 to + 1.423 V (vs. RHE) for the OER and from − 0.077 to 0.623 V for the HER. The scan rates for the HER and OER were 100 and 50 mV s^−1^, respectively. Details regarding the calculation of the electrochemically active surface area (EASA) are presented in Supplementary Material.

### Measurements of Liquid ZAB and All-Solid-State ZAB

In the rechargeable ZAB test, a Zn sheet (thickness of 0.5 mm) was used as the anode, the C cloth loaded with the catalyst was employed as the air–cathode, and 6 M KOH + 0.2 M ZnAc was used as the electrolyte. The procedure for preparing the catalyst ink was as follows. First, 3 mg of the sample was dispersed in 700 μL of a Nafion solution (70 μL of Nafion in 630 μL of absolute ethanol), followed by ultrasonication for 15 min. Subsequently, 600 μL of the catalyst was loaded on a C cloth, and the loading area was approximately 1 cm^2^. For the preparation of Pt/C + RuO_2_ as the control, 1.5 mg of commercial Pt/C and 1.5 mg of RuO_2_ were mixed in 630 μL of absolute ethanol, and then, 70 μL of Nafion was added. After 30 min of ultrasonication, 600 μL of the dispersion was employed for a control test. A ZAB test was performed at room temperature using a CHI-440 electrochemical workstation (CHI Instruments Inc.). LSV was conducted in the voltage range of 0.3–2.7 V at a scan rate of 10 mV s^−1^. The galvanostatic charge–discharge cycling curves were recorded at 10 mA cm^−2^ via chronopotentiometry, with 5 min of discharging and 5 min of charging. The electrochemical test method for the all-solid-state ZAB was similar to that for the liquid ZAB, with the differences being the electrolyte and the thickness of the Zn sheet (0.3 mm). The preparation method for the polyvinyl alcohol (PVA) electrolyte gel was as follows. Firstly, 666 mg of PVA-1788 and 333 mg of PVA-1799 were placed in a flask, and then, 10 mL of 6 M KOH + 0.2 M ZnAc was added. The mixture was kept under magnetic stirring at room temperature for 1 h and then transferred into an oil bath at 95 °C for 30 min. Subsequently, it was poured into a mold while being hot, frozen in a -70 °C refrigerator for 1 h, and finally removed and defrosted at 2 °C for 4 h to obtain the electrolyte gel. The catalyst loading was approximately 2 mg cm^−2^ for the all-solid-state ZAB test. The discharge power density was determined via LSV and calculated as Eq. 1 [47].

1 $${\text{Power}}\;{\text{density}}\;\left( {{\text{mW}}\;{\text{cm}}^{ - 2} } \right) = {\text{Voltage}} \times {\text{Current}}\;{\text{density}}$$

The specific capacity was determined using the galvanostatic discharge plot and calculated as Eq. 2.

2 $${\text{Specific capacity }}\left( {{\text{mAh g}}^{ - 1} } \right) = \frac{{{\text{Current}} \times {\text{Service hours}}}}{\text{Weight of consumed Zn}}.$$

## Results and Discussion

### Preparation of Ni_*x*_Co_1−*x*_Se Nanocages, Formation Mechanism, and Electron Microscopy

**Fig. 1:**
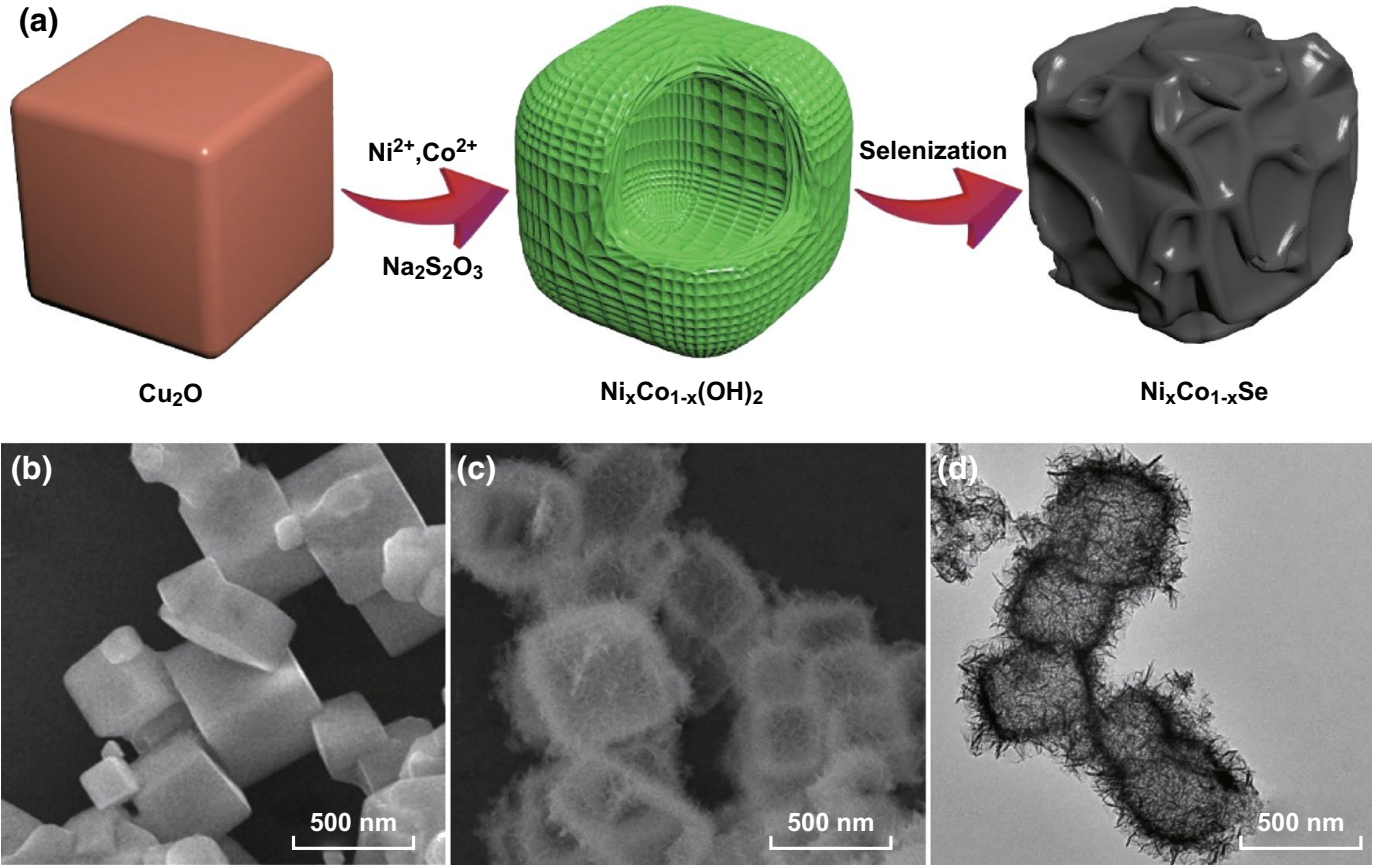
**a** Schematics of the fabrication process for the Ni_*x*_Co_1−*x*_Se nanocages. Representative SEM images of **b** the Cu_2_O cubes and **c** the Ni_0.2_Co_0.8_(OH)_2_ nanocages. **d** Representative TEM image of the Ni_0.2_Co_0.8_Se nanocages

Figure 1a shows schematics of the fabrication process for the Ni_*x*_Co_1−*x*_Se nanocages. Firstly, Cu_2_O nanocubes were synthesized by following a previously reported procedure [43]. Subsequently, in the presence of NaS_2_O_3_, the Cu_2_O reacted with Co^2+^ and Ni^2+^ ions to form NiCo hydroxide. After the selenization of the NiCo hydroxide, Ni_*x*_Co_1−*x*_Se nanocages were formed. SEM and TEM images of the Cu_2_O, Ni_0.2_Co_0.8_(OH)_2_, and Ni_0.2_Co_0.8_Se are shown in Fig. 1b–d. The Cu_2_O exhibited a well-defined cube shape, with some cubes clustered together. These well-defined cube morphologies are clearly recognized in Ni_0.2_Co_0.8_(OH)_2_, despite the numerous fine flocci appearing on the surface. To examine the structure of Ni_0.2_Co_0.8_(OH)_2_, we conducted XRD, energy-dispersive X-ray spectroscopy (EDX), and Fourier transform infrared (FT-IR) spectroscopic measurements (Fig. S1). The XRD results indicate that the Ni_0.2_Co_0.8_(OH)_2_ had an amorphous structure. Similar preparations and structures of Ni_*x*_Co_1−*x*_(OH)_2_ have been well documented [43, 48]. According to the EDX results, the atomic ratio of Ni to Co was 18.26:81.74, which is approximately 2:8. In the FT-IR spectrum, the broadband at 3447 cm^−1^ can be assigned to the stretching vibration of O–H groups, which were hydrogen-bonded to H_2_O molecules in the interlayer space. The band centered at 662 cm^−1^ is ascribed to *δ*(Ni–O–H), and the absorption band at 459 cm^−1^ is attributed to *υ*(Co/Ni–O) stretching vibrations [39]. Together, the results of XRD, EDX, and FT-IR confirm that Ni_0.2_Co_0.8_(OH)_2_ with an amorphous structure was successfully prepared. For Ni_0.2_Co_0.8_Se, hollow nanocages were observed, and their detailed surface structure was examined via HRTEM, as discussed later. The size evolution was monitored throughout the fabrication process, and the size distribution histograms are presented in Fig. S2. The average length of the Cu_2_O cubes, Ni_0.2_Co_0.8_(OH)_2_ nanocages, and Ni_0.2_Co_0.8_Se nanocages was approximately 509.2 ± 140.6, 573.8 ± 157.7, and 429.6 ± 85.1 nm, respectively, indicating that the size was not significantly changed by the chemical treatments as shown in Eqs. 3–7.

3 $${\text{Cu}}_{2} {\text{O}} + x{\text{S}}_{2} {\text{O}}_{3}^{2 - } + {\text{H}}_{2} {\text{O}} \to \left[ {{\text{Cu}}_{2} ({\text{S}}_{2} {\text{O}}_{3} )_{x} } \right]^{2 - 2x} + 2{\text{OH}}^{ - }$$

4 $$x{\text{Ni}}^{2 + } + \left( {1 - x} \right){\text{Co}}^{2 + } + 2{\text{OH}}^{ - } \to {\text{Ni}}_{x} {\text{Co}}_{1 - x} ({\text{OH}})_{2}$$

5 $$2{\text{SeO}}_{3}^{2 - } + {\text{CH}}_{2} {\text{OHCH}}_{2} {\text{OH}} \to 2{\text{Se}} + {\text{H}}_{2} {\text{C}}_{2} {\text{O}}_{4} + 4{\text{OH}}^{ - }$$

6 $$3{\text{Se}} + 6{\text{OH}}^{ - } \to 2{\text{Se}}^{2 - } + {\text{SeO}}_{3}^{2 - } + 3{\text{H}}_{2} {\text{O}}$$

7 $${\text{Se}}^{2 - } + {\text{Ni}}_{x} {\text{Co}}_{1 - x} ({\text{OH}})_{2} \to {\text{Ni}}_{x} {\text{Co}}_{1 - x} {\text{Se}} + 2{\text{OH}}^{ - }$$

A possible mechanism for the formation of hollow Ni_*x*_Co_1−*x*_Se nanocages is described as follows. According to the Pearson’s hard and soft acid–base principle, cuprous oxide can react with sodium thiosulfate to form a soluble complex, accompanied by the release of hydroxide ions (Eq. 3). In the presence of hydroxide ions, upon the introduction of Ni and Co ions, the immediately formed NiCo hydroxide precipitates can aggregate in situ, leading to the formation of Ni_*x*_Co_1−*x*_(OH)_2_ nanocages (Eq. 4). The selenization proceeds via an anion exchange mechanism. Under a high temperature and high pressure, EG can react with SeO_3_^2−^, generating elemental Se, hydroxide ions, and oxalic acid (Eq. 5). In the presence of hydroxide ions, the elemental Se can undergo the disproportionation reaction, forming SeO_3_^2−^ and Se^2−^ (Eq. 6). Finally, the generated Se^2−^ and the OH^−^ ions complete the anion exchange reaction, forming the Ni_*x*_Co_1−*x*_Se nanocages (Eq. 7).

**Fig. 2:**
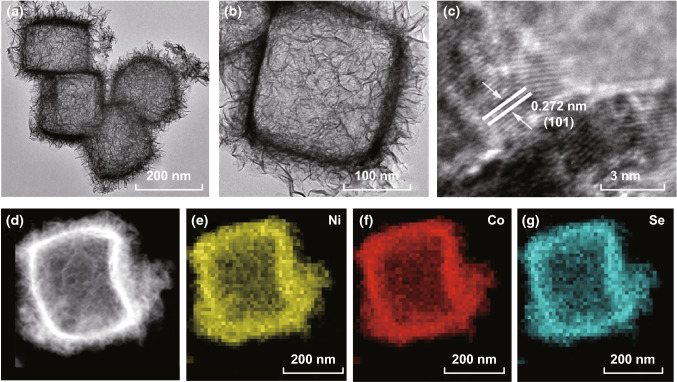
**a**–**c** Representative TEM and HRTEM images of the Ni_0.2_Co_0.8_Se nanocages. **d** HAADF-STEM image of Ni_0.2_Co_0.8_Se and **e**–**g** the corresponding elemental mapping images of Ni, Co, and Se

Figure 2a, b presents typical TEM images of the Ni_0.2_Co_0.8_Se nanocages with different magnifications. The hollow cube cage morphology is clearly observed. On the surface of the cages, there were numerous flocci, some of which were stacked or intersected together. According to the HRTEM image in Fig. 2c, the lattice spacing was 0.272 nm, which can be assigned to the crystal phase of NiCo (101). A representative high-angle annular dark-field imaging scanning TEM (HAADF-STEM) image of an Ni_0.2_Co_0.8_Se particle is shown in Fig. 2d, where a well-defined cage shape is clearly observed. The corresponding elemental mapping images of Ni, Co, and Se in Fig. 2e–g indicate that the three elements were homogeneously distributed with excellent uniformity, and all the elements had higher densities on the edges than in the core. These results confirm that the hollow nanocages of Ni_0.2_Co_0.8_Se were acquired via our designed strategy. Additionally, the atomic percentages were approximately determined using the EDX spectrum. As illustrated in Fig. S3, the calculated Ni–Co–Se atomic ratio was 10.03:38.90:50.07, which corresponds well to the initial loading molar ratio of 0.2:0.8:1.

### XRD and XPS Analyses

**Fig. 3:**
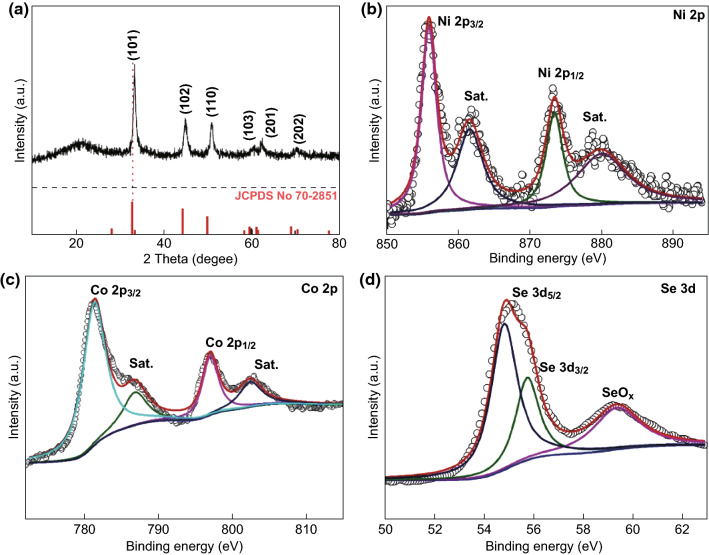
**a** XRD pattern of the Ni_0.2_Co_0.8_Se nanocages. The core-level XPS spectra for the **b** Ni 2*p*, **c** Co 2*p*, and **d** Se 3*d* electrons of Ni_0.2_Co_0.8_Se

XRD measurements were taken to further elucidate the crystal structure of the Ni_*x*_Co_1−*x*_Se samples. In Fig. 3a, the series of peaks at 32.9°, 44.6°, 50.3°, 59.6°, 61.5°, and 69.1° for Ni_0.2_Co_0.8_Se are in good accordance with the standard card of NiCoSe_2_ (JCPDS No. 70-2851), and these Bragg reflections can be assigned to the crystal phases of (101), (102), (110), (103), (201), and (202), respectively [39, 41, 49]. The XRD patterns of the other samples in the series, along with NiSe and CoSe, are shown in Fig. S4. Ni_0.5_Co_0.5_Se and Ni_0.8_Co_0.2_Se exhibit patterns similar to those of Ni_0.2_Co_0.8_Se, and the patterns for NiSe and CoSe agree well with the previously recorded feature [33, 34]. The peak of Ni_0.2_Co_0.8_Se is slightly offset from that of the standard card. As the Co ratio increases, the XRD peak position moves toward a larger diffraction angle, indicating that Co atoms were successfully doped into the Ni_0.5_Co_0.5_Se (same as the NiCoSe_2_ standard) lattice (Fig. S4). In contrast, the XRD peak of Ni_0.8_Co_0.2_Se had a smaller diffraction angle than that of Ni_0.5_Co_0.5_Se (Fig. S4). This strongly indicates the formation of Ni_0.2_Co_0.8_Se ternary compounds rather than a mixture of two solid phases. Because Ni_0.2_Co_0.8_Se, Ni_0.5_Co_0.5_Se, and Ni_0.8_Co_0.2_Se had the same hexagonal crystal structure and similar lattice parameters, the Ni_0.5_Co_0.5_Se compound was intentionally prepared as a reference, and its diffraction pattern fully matched the standard card of JCPDS No. 70-2851. The results indicate that the designed method for preparing the compounds was rational and successful. Such subtle manipulation of the lattice through the optimization of the substrate elements has been previously reported [33, 43].

Subsequently, the chemical states of the composites were investigated via XPS, and the spectra are shown in Fig. 3b–d. In Fig. 3b, the two striking peaks with binding energies of 873.1 and 855.2 eV and the two satellite peaks can be assigned to the Ni 2*p*_1/2_ and Ni 2*p*_3/2_ electrons, respectively, strongly indicating that elemental Ni existed as Ni(II) [39]. In Fig. 3c, the Co 2*p*_1/2_ and Co 2*p*_3/2_ signals (797.4 and 781.3 eV) and two satellite peaks are characteristics of Co(II) [39]. The high-resolution Se 3*d* spectra can be deconvoluted into two peaks at 55.6 and 54.8 eV, which correspond well to the Se 3*d*_3/2_ and Se 3*d*_5/2_ electrons, respectively. Interestingly, the peak at 59.3 eV indicates the formation of SeO_*x*_, which was probably due to the surface oxidation of selenide [49].

The high-resolution XPS spectra of the Ni 2*p*, Co 2*p*, and Se 3*d* electrons of Ni_0.5_Co_0.5_Se, Ni_0.8_Co_0.2_Se, NiSe, and CoSe are shown in Fig. S5. In the Ni 2*p* spectrum (S5c1) and the NiSe and Co 2*p* (S5d1) spectrum for CoSe, the binding energies correspond to the Ni(II) and Co(II) species. For Ni_0.5_Co_0.5_Se, there are two distinctive peaks at 855.6 and 873.4 eV in the Ni 2*p* spectrum (S5a1), and both binding energy values correspond to the chemical valences exhibited by the Ni element in Ni_0.2_Co_0.8_Se, suggesting the presence of Ni(II). In the Co 2*p* spectrum (S5a2), the two sharp peaks (Co 2*p*_3/2_ and Co 2*p*_1/2_) at 781.2 and 797.9 eV are attributed to the Co(II) species. For Ni_0.8_Co_0.2_Se, the core-level Ni 2*p* spectrum (S5b1) exhibits two peaks at 855.4 and 873.3 eV, which are indexed to the Ni 2*p*_3/2_ and Ni 2*p*_1/2_ electrons, respectively, and there are two corresponding shakeup satellite peaks at 861.6 and 880.1 eV. The Co 2*p* spectrum (S5b2) exhibits a similar feature to the Co 2*p* spectrum for CoSe, implying that the Co exists as Co(II). Furthermore, the core-level Se 3*d* spectra (S5a3, b3, c2, and d2) of Ni_0.5_Co_0.5_Se, Ni_0.8_Co_0.2_Se, NiSe, and CoSe all exhibit two distinct characteristic peaks around 55.1 and 59.3 eV. The peak at 55.1 eV can be fitted into two sub-peaks representing the Se 3*d*_5/2_ and Se 3*d*_3/2_ electrons from the Se element. The other peak at 59.3 eV is probably due to the oxidation of surface Se and the formed Se–O bonds [49].

### OER Performance

**Fig. 4:**
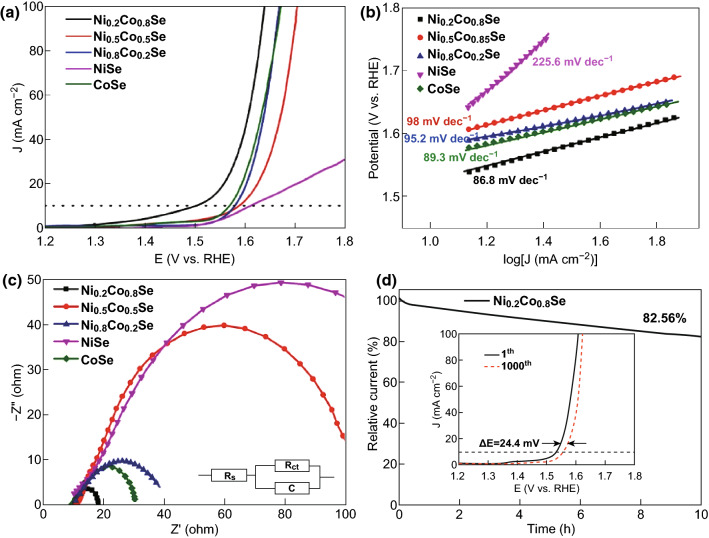
**a** IR-corrected LSV curves of Ni_0.2_Co_0.8_Se, Ni_0.5_Co_0.5_Se, Ni_0.8_Co_0.2_Se, NiSe, and CoSe in an N_2_-saturated 1 M KOH solution obtained at a scan rate of 10 mV s^−1^. **b** Tafel plots of the Ni_0.2_Co_0.8_Se, Ni_0.5_Co_0.5_Se, Ni_0.8_Co_0.2_Se, NiSe, and CoSe catalysts for the OER in 1.0 M KOH. **c** EIS Nyquist plots, where Rs represents the electrolyte resistance, C represents the double-layer capacitance, and *R*_ct_ represents the charge-transfer resistance. **d** Chronoamperometric response of Ni_0.2_Co_0.8_Se nanocages. The inset shows the LSV curves of an Ni_0.2_Co_0.8_Se electrode tested after 1 and 1000 CV cycles

The electrochemical properties of the Ni_*x*_Co_1−*x*_Se series toward the OER were examined, and the electrocatalytic performance is compiled in Table 1. Figure 4a shows the LSV curves of the Ni_*x*_Co_1−*x*_Se series, NiSe, and CoSe tested in N_2_-saturated 1 M KOH. With the decrease in the Ni percentage in the total transition metal, the OER activity gradually intensified. The Ni_0.2_Co_0.8_Se sample exhibited the best activity. NiSe had negligible OER activity, whereas the performance of CoSe was only slightly inferior to that of Ni_0.2_Co_0.8_Se. For obtained current density of 10 mA cm^−2^, the required overpotential was 280, 360, 350, and 345 mV for Ni_0.2_Co_0.8_Se, Ni_0.5_Co_0.5_Se, Ni_0.8_Co_0.2_Se, and CoSe, respectively. Ni_0.2_Co_0.8_Se exhibited the best OER activity in the series, and its OER activity was markedly superior to that of the benchmark IrO_2_ catalyst for the OER (Fig. S6, overpotential of 354 mV at 10 mA cm^−2^). The corresponding Tafel plots are presented in Fig. 4b, where the Tafel slope can be extrapolated and calculated. As expected, NiSe exhibited the largest slope of 225.6 mA cm^−1^, owing to the sluggish reaction kinetics. The Tafel slope was 86.8, 98, 95.2, and 89.3 mV dec^−1^ for Ni_0.2_Co_0.8_Se, Ni_0.5_Co_0.5_Se, Ni_0.8_Co_0.2_Se, and CoSe, respectively. Ni_0.2_Co_0.8_Se had the lowest value in the series, which was lower than that of the benchmark IrO_2_ catalyst (117.6 mV dec^−1^), indicating fast reaction kinetics. Electrochemical impedance spectroscopy (EIS) was then conducted, as shown in Fig. 4c. NiSe exhibited a near-straight line in the wide potential widow, in good accordance with its weak OER activity. Among the Ni_*x*_Co_1−*x*_Se compounds, Ni_0.2_Co_0.8_Se exhibited the smallest semicircle, indicating that it had the lowest electron-transfer resistance. Lastly, the long-term stability of Ni_0.2_Co_0.8_Se toward the OER was examined, as shown in Fig. 4d. According to the chronoamperometric *i*–*t* curve, 82.56% of the initial current was retained after continuous testing for 10 h. Additionally, as shown in the inset of Fig. 4d, after 1000 cycles of potential scans, an extremely low additional overpotential of 24.4 mV was needed to obtain a current density of 10 mA cm^−2^.

**Table 1:** OER and HER activity for the Ni_*x*_Co_1−*x*_Se series, including the results of OER and HER tests in 1 M KOH, as well as the electrochemical properties

Sample	OER (1 M KOH)	HER (1 M KOH)	*C*_DL_ (mF)	EASA (cm^2^)
	Overpotential @ 10 mA cm^−2^ (mV)	Overpotential @10 mA cm^−2^ (mV)		
Ni_0.2_Co_0.8_Se	280	73	24.13	603.25
Ni_0.5_Co_0.5_Se	360	112	1.87	46.75
Ni_0.8_Co_0.2_Se	350	93	2.62	65.50
NiSe	390	150	0.52	13.00
CoSe	345	99	3.13	78.25

### HER Performance

**Fig. 5:**
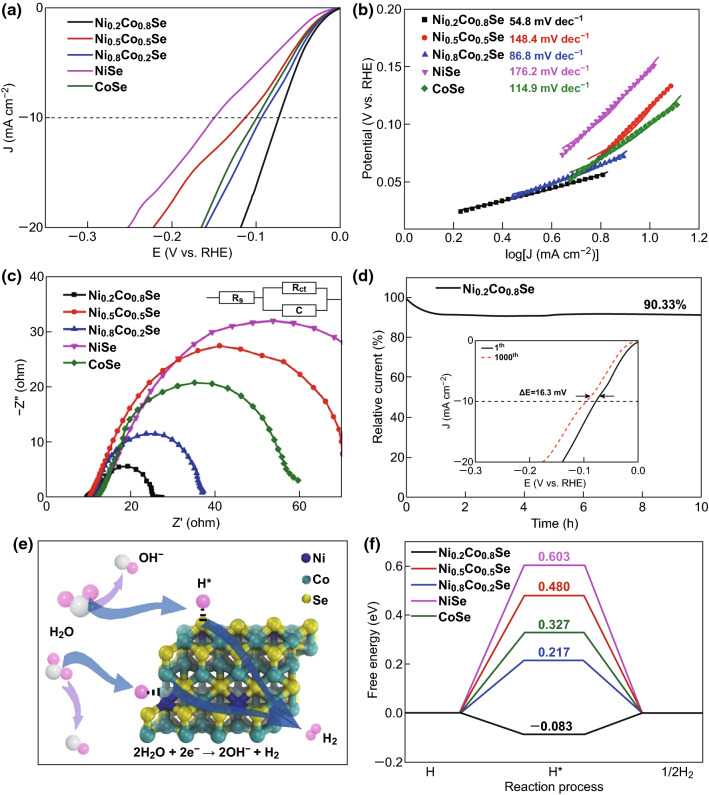
**a** HER polarization curves of Ni_0.2_Co_0.8_Se, Ni_0.5_Co_0.5_Se, Ni_0.8_Co_0.2_Se, NiSe, and CoSe obtained at a scan rate of 10 mV s^−1^ in 1.0 M KOH. **b** Corresponding Tafel plots. **c** EIS Nyquist plots, where *R*_s_ represents the electrolyte resistance, *C* represents the double-layer capacitance, and *R*_ct_ represents the charge-transfer resistance. **d** HER polarization curves of the Ni_0.2_Co_0.8_Se electrode tested after 1 and 1000 CV cycles. The inset shows the chronoamperometric response of Ni_0.2_Co_0.8_Se at 10 mA cm^−2^. **e** Schematic of the HER at the Ni_0.2_Co_0.8_Se active interface in the alkaline environment. **f** Adsorption free energy diagram for the Volmer reaction steps

Next, the Ni_*x*_Co_1−*x*_Se samples were subjected to an HER test (Table 1). Figure 5a presents the LSV curves. To obtain a current density of 10 mA cm^−2^, the required overpotential was 73, 112, 93, 150, and 99 mV for Ni_0.2_Co_0.8_Se, Ni_0.5_Co_0.5_Se, Ni_0.8_Co_0.2_Se, NiSe, and CoSe, respectively. NiSe exhibited the weakest catalytic activity, and the performance of CoSe was inferior to that of Ni_0.8_Co_0.2_Se and Ni_0.2_Co_0.8_Se, in contrast to the results of the OER test. The best HER performance was exhibited by the Ni_0.2_Co_0.8_Se sample, whose activity was close to that of the benchmark Pt/C catalyst for the HER (Fig. S7, overpotential of 38.1 mV at 10 mA cm^−2^). The Tafel plots of the samples are presented in Fig. 5b, and the Tafel slopes were calculated. The Tafel slope was 54.8, 148.4, 86.8, 176.2, and 114.9 mV dec^−1^ for Ni_0.2_Co_0.8_Se, Ni_0.5_Co_0.5_Se, Ni_0.8_Co_0.2_Se, NiSe, and CoSe, respectively. This trend matches the aforementioned overpotential values. Ni_0.2_Co_0.8_Se exhibited the lowest Tafel slope value, indicating that it had the fastest reaction kinetics. Its Tafel slope is close to that of Pt/C (40.3 mV dec^−1^), suggesting that a Tafel–Volmer mechanism occurred and that the rate-determining step in the HER was probably the electrochemical desorption of H_2_ [50, 51]. Figure 5c shows the electrochemical impedance spectra of the samples. As anticipated, NiSe exhibited the largest semicircle. Ni_0.2_Co_0.8_Se exhibited the smallest semicircle, indicating that it had the lowest electron-transfer resistance, which agrees well with its high HER activity. Finally, the long-term stability of the samples was tested via both *i*–*t* measurements and an ADT. As illustrated in Fig. 5d, after continuous testing for approximately 10 h, 90.33% of the initial current was retained in the chronoamperometric measurement. The inset shows that after 1000 cycles of potential scans, at the current density of 10 mA cm^−2^, the overpotential only shifted by 16.3 mV. Both tests indicate the robust stability of the Ni_0.2_Co_0.8_Se sample in the long-term operation for the HER. To determine the reason for the high activity of Ni_*x*_Co_1−*x*_Se toward the HER, the Gibbs free energy of H adsorption was calculated using the Norskov scheme (see details in Supplementary Material). First, the H adsorption free energy on the surface of pure NiSe(101) and CoSe(101) was calculated. Then, to elucidate the interplay between the Ni and Co dopants, models of the Ni-doped CoSe bulk material and the Co-doped NiSe bulk material with corresponding Co-to-Ni ratios were constructed, and all possible adsorption sites on the (101) planes were examined. Figure 5e illustrates the HER principle for the theoretical calculations under alkaline conditions, accompanied by the active sites of Ni_0.2_Co_0.8_Se. The active sites of the other samples are shown in Fig. S8. According to the reaction pathways, the enthalpies of the rate-determining Volmer step on the Ni_*x*_Co_1−*x*_Se (101) surfaces were determined. For hydrogen adsorption, as shown in Fig. 5f, Ni_0.2_Co_0.8_Se exhibited the lowest Gibbs free energy among the samples. The simulation results are in good accordance with the experimental results, providing theoretical evidence that Ni_0.2_Co_0.8_Se has the best HER performance among the samples in the series.

### EASA Analysis and ORR Performance

The OER and HER performance of the Ni_0.2_Co_0.8_Se sample is comparable, if not superior, to that of most recently reported transition metal selenide-based materials, and the comparison results are presented in Table S1. For instance, in 1 M KOH for the OER, to obtain a current density of 10 mA cm^−2^, the required overpotential of Ni_0.2_Co_0.8_Se was 280 mV, which is lower than those for CoSe_2_/Mn_3_O_4_ (450 mV) [52], Ni_*x*_Se (330 mV) [33], Co_0.85_Se (320 mV) [34], and NiSe_2_/Ti (295 mV) [53] and comparable to those for Co(S_0.22_Se_0.78_)_2_ (283 mV) [54], Ni_0.75_Fe_0.25_Se_2_ (272 mV) [36], and NiSe/NF (270 mV) [55]. In the HER test, at 10 mA cm^−2^, the overpotential was 73 mV for Ni_0.2_Co_0.8_Se, which is significantly lower than those for Ni_*x*_Se (233 mV) [33], Co_0.85_Se (230 mV) [34], Co(S_0.22_Se_0.78_)_2_ (175 mV) [54], and NiSe/NF (96 mV) [55] and comparable to that for NiSe_2_/Ti (70 mV) [53] under the same conditions. These comparison results indicate that Ni_0.2_Co_0.8_Se is a superior multifunctional catalyst for the OER and HER.

To determine the reason for the difference in electrocatalytic activity among the samples, EASA measurements were taken [43]. The EASA values were estimated according to the electrochemical double-layer capacitance (*C*_DL_) of the catalyst, and the *C*_DL_ was measured via cyclic voltammograms (Fig. S9) within a potential range where no apparent Faradaic process occurred. The detailed calculations are presented in Supplementary Material, and the calculation results are presented in Table 1. The EASA values well explain the trend of the electrocatalytic performance, as they are in good accordance with the OER activity order of the series (Ni_0.2_Co_0.8_Se > CoSe > Ni_0.8_Co_0.2_Se > Ni_0.5_Co_0.5_Se > NiSe). The EASA of Ni_0.2_Co_0.8_Se was the largest among the samples and was approximately 10 times larger than those of Ni_0.8_Co_0.2_Se and Ni_0.5_Co_0.5_Se.

In addition, the ORR performance of the Ni_*x*_Co_1−*x*_Se series was evaluated in an alkaline solution. Figure S10 shows the LSV polarization curves obtained with a rotation rate of 1600 rpm in 0.1 M KOH. The ORR activity matched the trend of the EASA values. As expected, the Ni_0.2_Co_0.8_Se sample had the best activity in the series. Its onset potential was 0.87 V, and its diffusion-limiting current density was 4.45 mA cm^−2^. Although its half-wave potential (0.769 V) was inferior to that of the commercial Pt/C (0.86 V), its limiting current density was higher than that of Pt/C (4.32 mA cm^−2^). A large anode peak appeared at approximately 1.0 V, which was mainly due to the oxidation of the metal ions. A corresponding reduction peak appeared at approximately 0.7 V, which is ascribed to the reduction of Ni^3+^ (Co^3+^) to Ni^2+^ (Co^2+^) [56]. Additionally, there was a broad peak caused by the oxidation or reduction of surface Se, which was observed in previous studies [57, 58].

### ZAB Performance

Inspired by the excellent performance of Ni_0.2_Co_0.8_Se toward the OER and HER, we investigated its practical applicability by employing it as a multifunctional electrocatalyst for a ZAB and for overall water splitting. First, a proof-of-concept liquid ZAB was assembled (Fig. S11), which comprised Ni_0.2_Co_0.8_Se as the air–cathode, a Zn plate as the anode, and 0.2 M ZnAc + 6 M KOH as the electrolyte [51, 59]. The reactions between the anode and the cathode during the charging and discharging of the ZAB are expressed as Eqs. 8–12 [60].

8 $${\text{Anode}}:{\text{Zn}} + 4{\text{OH}}^{ - } \to {\text{Zn}}\left( {\text{OH}} \right)_{4}^{2 - } + 2{\text{e}}^{ - }$$

9 $${\text{Zn}}\left( {\text{OH}} \right)_{4}^{2 - } \to {\text{ZnO}} + {\text{H}}_{2} {\text{O}} + 2{\text{OH}}^{ - }$$

10 $${\text{Cathode}}:{\text{O}}_{2} + 4{\text{e}}^{ - } + 2{\text{H}}_{2} {\text{O}} \to 4{\text{OH}}^{ - }$$

11 $${\text{Overall}}\,{\text{reaction}}: \, 2{\text{Zn}} + {\text{O}}_{2} \to \, 2{\text{ZnO}}$$

12 $${\text{Parasitic}}\,{\text{reaction}}:{\text{ Zn}} + 2{\text{H}}_{2} {\text{O}} \to {\text{Zn}}\left( {\text{OH}} \right)_{2} + {\text{H}}_{2}$$

**Fig. 6:**
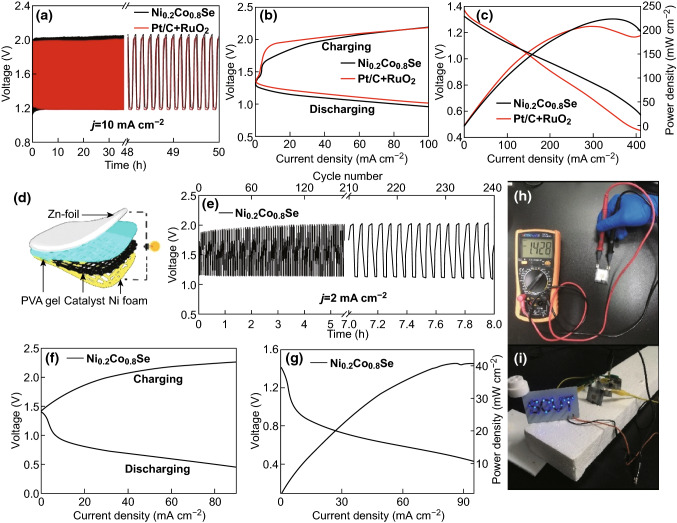
**a** Cycling performance of the rechargeable ZAB using Ni_0.2_Co_0.8_Se at 10 mA cm^−2^, where each cycle lasted 5 min. **b** Charge–discharge polarization curves of the rechargeable ZABs using Ni_0.2_Co_0.8_Se and Pt/C + RuO_2_. **c** Polarization and power density curves of the ZABs using the Ni_0.2_Co_0.8_Se and Pt/C + RuO_2_ catalysts. **d** Schematic of the self-made all-solid-state ZAB. **e** Cycling performance of the all-solid-state ZAB using Ni_0.2_Co_0.8_Se at 2 mA cm^−2^, where each cycle lasted 2 min. **f** Charge–discharge polarization curves of the all-solid-state ZAB using Ni_0.2_Co_0.8_Se. **g** Polarization and power density curves of the all-solid-state ZAB using Ni_0.2_Co_0.8_Se. **h** Open-circuit voltage of an all-solid-state ZAB and **i** an LED circuit board powered by three all-solid-state ZABs

The ZAB based on the Ni_0.2_Co_0.8_Se air–cathode exhibited a stable open-circuit voltage of 1.44 V, which is close to that of the Pt/C + RuO_2_ electrode (1.46 V) (Fig. S12), indicating that its performance is at least comparable to that of the conventional precious metal-based Pt/C + RuO_2_ catalyst. The Ni_0.2_Co_0.8_Se exhibited the highest open-circuit potential in the series (Fig. S12). The undulating charge–discharge voltages of Ni_0.2_Co_0.8_Se and Pt/C + RuO_2_ are shown in Fig. 6a. With continuous testing for over 50 h, the voltage gap of Ni_0.2_Co_0.8_Se remained at approximately 0.873 V, which is comparable to that of the Pt/C + RuO_2_ catalyst (0.845 V). For Ni_0.2_Co_0.8_Se, the initial round-trip efficiency at 5 h was 61.04%, and after 50 h of constant current charge–discharge cycles, the round-trip efficiency was 60.86% with no attenuation of the performance, which is superior to that of Pt/C + RuO_2_ (58.91%) at 50 h. Furthermore, as indicated by the galvanostatic charge–discharge curves of the Ni_*x*_Co_1−*x*_Se samples (Fig. S13a), only Ni_0.2_Co_0.8_Se maintained a high stability and minimal voltage gap after 50 h of continuous operation. Thus, Ni_0.2_Co_0.8_Se has excellent charge–discharge performance and stability. Figure 6b shows the charge–discharge polarization curves of rechargeable ZABs using Ni_0.2_Co_0.8_Se and Pt/C + RuO_2_. Ni_0.2_Co_0.8_Se (0.9436 V) exhibited a smaller voltage gap than Pt/C + RuO_2_ (0.9638 V) at the current density of 50 mA cm^−2^, indicating that it had a higher charge–discharge capacity. The charge–discharge polarization curves (Fig. S13b) of Ni_0.5_Co_0.5_Se, Ni_0.8_Co_0.2_Se, NiSe, and CoSe show that the voltage gaps were 1.160, 1.186, 1.347, and 1.157 V, respectively, at the current density of 50 mA cm^−2^. Ni_0.2_Co_0.8_Se exhibited the smallest voltage gap, indicating its excellent charge–discharge performance. Moreover, the discharge and corresponding power density curves are presented in Fig. 6c. Ni_0.2_Co_0.8_Se had a high power density of 223.5 mW cm^−2^, which is higher than that of the Pt/C + RuO_2_ catalyst (210.4 mW cm^−2^), indicating its superiority for practical ZAB applications. Figure S13c shows that the maximum power density of Ni_0.5_Co_0.5_Se, Ni_0.8_Co_0.2_Se, NiSe, and CoSe was 153.8, 160.3, 134.4, and 181.3 mW cm^−2^, respectively. All of these values are lower than that of Ni_0.2_Co_0.8_Se. Thus, Ni_0.2_Co_0.8_Se had the highest discharge power density in the Ni_*x*_Co_1−*x*_Se series. Figure S13d depicts the typical galvanostatic discharge profile at the current density of 10 mA cm^−2^ with Ni_*x*_Co_1−*x*_Se as the air–cathode. The specific capacity normalized to the weight of the consumed Zn plate was 698.6, 685.9, 664.8, 553.1, and 620.5 mAh g^−1^ for Ni_0.2_Co_0.8_Se, Ni_0.5_Co_0.5_Se, Ni_0.8_Co_0.2_Se, NiSe, and CoSe, respectively. Ni_0.2_Co_0.8_Se exhibited the best performance. Given the excellent performance of Ni_0.2_Co_0.8_Se in the primary liquid ZAB, a portable, simple, and industrialized all-solid-state ZAB was fabricated, and the performance of Ni_0.2_Co_0.8_Se in this device was examined. Figure 6d shows a schematic of the all-solid-state ZAB, and the fabrication steps are presented in Fig. S14. Figure 6e shows that the all-solid-state ZAB had a significant cycle life with an initial voltage gap of 0.71 V. After 240 cycles, the round-trip efficiency was attenuated from 61.96 to 55.26%. The efficiency decay of only 6.7% confirms the excellent stability of the all-solid-state ZAB using Ni_0.2_Co_0.8_Se. Owing to the high contact resistance of the battery components and the poor conductivity of the sand-absorbing alkaline PVA electrolyte, the all-solid-state ZAB was slightly less efficient than the liquid ZAB [61]. The charging and discharging polarization curves of the all-solid-state ZAB are presented in Fig. 6f. At the current density of 20 mA cm^−2^, the voltage gap was 0.87 V, indicating outstanding charging and discharging performance. Figure 6g shows the polarization curves. The power density of the Ni_0.2_Co_0.8_Se-modified all-solid-state ZAB was calculated as 41.03 mW cm^−2^. This battery also exhibited an impressive open-circuit potential of 1.428 V (Fig. 6h). Finally, we connected three all-solid-state ZABs in series with an open-circuit voltage of approximately 4.36 V (Fig. S15) and thus powered a board that illuminated light-emitting diodes (LEDs) with “SCUT” symbols (Fig. 6i).

The performance of Ni_0.2_Co_0.8_Se in both the liquid ZAB and the all-solid-state ZAB was superior to that of the recently reported Co-based nanostructures. The comparison results are presented in Table S2. In the liquid ZAB test under the same conditions, the open-circuit potential for Ni_0.2_Co_0.8_Se was higher than those for Co-NDC [62] and NGM-Co [63], and the power density was higher than those for CoN_4_/NG [64], NGM-Co [63], and Co-NDC [62]. In the all-solid-state ZAB test, the open-circuit potential for Ni_0.2_Co_0.8_Se was higher than those for NC-Co/CoN_*x*_ [65], Co-NDC [62], and Co_3_O_4_/N-rGO [29]; the round-trip efficiency was higher than that for CoN_4_/NG [64]; and the power density was higher than those for CoN_4_/NG [64], NGM-Co [63], and Co_3_O_4_/N-rGO [29]. The outstanding performance of Ni_0.2_Co_0.8_Se in the ZAB test is largely attributed to its excellent electrocatalytic performance, as discussed previously.

### Overall Water-Splitting Test

**Fig. 7:**
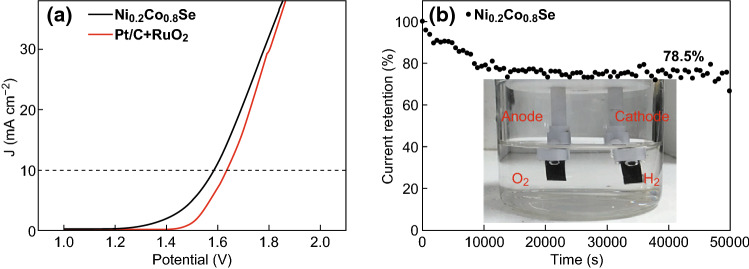
**a** Water-splitting polarization curves of Ni_0.2_Co_0.8_Se and Pt/C + RuO_2_ in 1 M KOH. **b** Chronoamperometric response of Ni_0.2_Co_0.8_Se nanocages for overall water splitting. The inset shows the gas evolution on the two electrodes at 10 mA cm^−2^

Next, Ni_0.2_Co_0.8_Se was employed for overall water splitting in an alkalic solution, in comparison with the Pt/C + RuO_2_ catalyst [51, 66, 67]. Ni_0.2_Co_0.8_Se was used as the catalyst in both the cathode and the anode. In a control experiment, Pt/C was used as the cathode catalyst, and RuO_2_ was employed as the anode catalyst. Figure 7a presents the water-splitting polarization curves of Ni_0.2_Co_0.8_Se and Pt/C + RuO_2_ in a 1 M KOH solution. For obtaining a current density of 10 mA cm^−2^, the required cell voltage was 1.592 V for Ni_0.2_Co_0.8_Se, which is significantly lower than that for the Pt/C + RuO_2_ catalyst (1.628 V). Thus, Ni_0.2_Co_0.8_Se had a better water-splitting capability than the combined precious metal-based standard catalyst in the alkaline solution. The long-term durability of the Ni_0.2_Co_0.8_Se sample was evaluated via chronoamperometric measurement for 50,000 s. As shown in Fig. 7b, after constant water-splitting operation for approximately 13 h, Ni_0.2_Co_0.8_Se retained 78.5% of its initial current. The inset shows that H_2_ and O_2_ bubbles were visible at the cathode and anode, respectively. These findings confirm that Ni_0.2_Co_0.8_Se is a promising high-efficiency, cost-effective electrocatalyst for overall water-splitting devices in alkaline solutions.

## Conclusions

We demonstrated the facile fabrication of a series of Ni_*x*_Co_1−*x*_Se samples with well-defined cages and investigated their catalytic performance for OER, HER, and ORR electrocatalysis. Among the Ni_*x*_Co_1−*x*_Se compounds, Ni_0.2_Co_0.8_Se exhibited the best performance, as indicated by the lowest overpotential of 280 and 73 mV to obtain a current density of 10 mA cm^−2^ for the OER and HER, respectively. Moreover, Ni_0.2_Co_0.8_Se was engineered as an air–cathode of both a rechargeable ZAB and an all-solid-state ZAB and employed as a catalyst for overall water splitting. It endowed both ZAB devices with outstanding performance, including a long cycling lifetime, high round-trip efficiency, and high power density, and achieved total water splitting with excellent efficiency at a low cell voltage. The study paves a pathway for preparing transition metal selenides with a well-defined morphology and optimized stoichiometric ratio as promising catalysts for renewable energy technologies, such as rechargeable and all-solid-state metal–air batteries and water-splitting devices.

## Electronic supplementary material

Below is the link to the electronic supplementary material.

Supplementary material 1 (PDF 1328 kb)

## References

[1] Li Y, Dai H (2014). Recent advances in zinc-air batteries. Chem. Soc. Rev..

[2] Yin J, Li Y, Lv F, Lu M, Sun K, Wang W (2017). Oxygen vacancies dominated NiS_2_/CoS_2_ interface porous nanowires for portable Zn–air batteries driven water splitting devices. Adv. Mater..

[3] Chen X, Zhang Z, Chi L, Nair AK, Shangguan W, Jiang Z (2016). Recent advances in visible-light-driven photoelectrochemical water splitting: catalyst nanostructures and reaction systems. Nano-Micro Lett..

[4] Zhao Q, Yan Z, Chen C, Chen J (2017). Spinels: controlled preparation, oxygen reduction/evolution reaction application, and beyond. Chem. Rev..

[5] Liang K, Guo L, Marcus K, Zhang S, Yang Z (2017). Overall water splitting with room-temperature synthesized nife oxyfluoride nanoporous films. ACS Catal..

[6] Li J, Xu W, Luo J, Zhou D, Zhang D, Wei L, Xu P, Yuan D (2017). Synthesis of 3d hexagram-like cobalt–manganese sulfides nanosheets grown on nickel foam: a bifunctional electrocatalyst for overall water splitting. Nano-Micro Lett..

[7] Han X, Wu X, Deng Y, Liu J, Lu J, Zhong C, Hu W (2018). Ultrafine pt nanoparticle-decorated pyrite-type CoS_2_ nanosheet arrays coated on carbon cloth as a bifunctional electrode for overall water splitting. Adv. Energy Mater..

[8] Liu M, Zhang R, Chen W (2014). Graphene-supported nanoelectrocatalysts for fuel cells: synthesis, properties, and applications. Chem. Rev..

[9] Shao M, Chang Q, Dodelet J-P, Chenitz R (2016). Recent advances in electrocatalysts for oxygen reduction reaction. Chem. Rev..

[10] Wang L, Tang Z, Yan W, Wang Q, Yang H, Chen S (2017). Co@pt core@shell nanoparticles encapsulated in porous carbon derived from zeolitic imidazolate framework 67 for oxygen electroreduction in alkaline media. J. Power Sour..

[11] Strmcnik D, Lopes PP, Genorio B, Stamenkovic VR, Markovic NM (2016). Design principles for hydrogen evolution reaction catalyst materials. Nano Energy.

[12] Wang J, Xu F, Jin H, Chen Y, Wang Y (2017). Non-noble metal-based carbon composites in hydrogen evolution reaction: fundamentals to applications. Adv. Mater..

[13] Seitz LC, Dickens CF, Nishio K, Hikita Y, Montoya J (2016). A highly active and stable IrO_*x*_/SrIrO_3_ catalyst for the oxygen evolution reaction. Science.

[14] Reier T, Oezaslan M, Strasser P (2012). Electrocatalytic oxygen evolution reaction (OER) on Ru, Ir, and Pt catalysts: a comparative study of nanoparticles and bulk materials. ACS Catal..

[15] Niu W, Li L, Liu X, Wang N, Liu J, Zhou W, Tang Z, Chen S (2015). Mesoporous *n*-doped carbons prepared with thermally removable nanoparticle templates: an efficient electrocatalyst for oxygen reduction reaction. J. Am. Chem. Soc..

[16] Wu G, Wang J, Ding W, Nie Y, Li L, Qi X, Chen S, Wei Z (2016). A strategy to promote the electrocatalytic activity of spinels for oxygen reduction by structure reversal. Angew. Chem. Int. Ed..

[17] Xu ZJ (2017). From two-phase to three-phase: the new electrochemical interface by oxide electrocatalysts. Nano-Micro Lett..

[18] Wang H, Yang N, Li W, Ding W, Chen K (2018). Understanding the roles of nitrogen configurations in hydrogen evolution: trace atomic cobalt boosts the activity of planar nitrogen-doped graphene. ACS Energy Lett..

[19] Xu L, Jiang Q, Xiao Z, Li X, Huo J, Wang S, Dai L (2016). Plasma-engraved Co_3_O_4_ nanosheets with oxygen vacancies and high surface area for the oxygen evolution reaction. Angew. Chem. Int. Ed..

[20] Niu W, Pakhira S, Marcus K, Li Z, Mendoza-Cortes JL, Yang Y (2018). Apically dominant mechanism for improving catalytic activities of n-doped carbon nanotube arrays in rechargeable zinc–air battery. Adv. Energy Mater..

[21] Lei C, Chen H, Cao J, Yang J, Qiu M (2018). Fe-n_4_ sites embedded into carbon nanofiber integrated with electrochemically exfoliated graphene for oxygen evolution in acidic medium. Adv. Energy Mater..

[22] Chen D, Qiao M, Lu Y-R, Hao L, Liu D, Dong C-L, Li Y, Wang S (2018). Preferential cation vacancies in perovskite hydroxide for the oxygen evolution reaction. Angew. Chem. Int. Ed..

[23] Wang Y, Xie C, Zhang Z, Liu D, Chen R, Wang S (2018). In situ exfoliated, n-doped, and edge-rich ultrathin layered double hydroxides nanosheets for oxygen evolution reaction. Adv. Funct. Mater..

[24] Dou S, Wang X, Wang S (2018). Rational design of transition metal-based materials for highly efficient electrocatalysis. Small Methods.

[25] Song Z, Han X, Deng Y, Zhao N, Hu W, Zhong C (2017). Clarifying the controversial catalytic performance of Co(OH)_2_ and Co_3_O_4_ for oxygen reduction/evolution reactions toward efficient Zn–air batteries. ACS Appl. Mater. Interfaces.

[26] Chen X, Liu B, Zhong C, Liu Z, Liu J (2017). Ultrathin co_3_o_4_ layers with large contact area on carbon fibers as high-performance electrode for flexible zinc–air battery integrated with flexible display. Adv. Energy Mater..

[27] Yang Z, Zhang J-Y, Liu Z, Li Z, Lv L (2017). “Cuju”-structured iron diselenide-derived oxide: a highly efficient electrocatalyst for water oxidation. ACS Appl. Mater. Interfaces..

[28] Zhang J-Y, Wang H, Tian Y, Yan Y, Xue Q (2018). Anodic hydrazine oxidation assists energy-efficient hydrogen evolution over a bifunctional cobalt perselenide nanosheet electrode. Angew. Chem. Int. Ed..

[29] Li Y, Zhong C, Liu J, Zeng X, Qu S (2018). Atomically thin mesoporous Co_3_O_4_ layers strongly coupled with n-rgo nanosheets as high-performance bifunctional catalysts for 1D knittable zinc–air batteries. Adv. Mater..

[30] Liu Y, Cheng H, Lyu M, Fan S, Liu Q (2014). Low overpotential in vacancy-rich ultrathin CoSe_2_ nanosheets for water oxidation. J. Am. Chem. Soc..

[31] Sivanantham A, Shanmugam S (2017). Nickel selenide supported on nickel foam as an efficient and durable non-precious electrocatalyst for the alkaline water electrolysis. Appl. Catal. B Environ..

[32] Liu Z-Q, Cheng H, Li N, Ma TY, Su YZ (2016). ZnCo_2_O_4_ quantum dots anchored on nitrogen-doped carbon nanotubes as reversible oxygen reduction/evolution electrocatalysts. Adv. Mater..

[33] Zheng X, Han X, Liu H, Chen J, Fu D, Wang J, Zhong C, Deng Y, Hu W (2018). Controllable synthesis of nixse (0.5 ≤ *x* ≤ 1) nanocrystals for efficient rechargeable zinc–air batteries and water splitting. ACS Appl. Mater. Interfaces..

[34] Meng T, Qin J, Wang S, Zhao D, Mao B, Cao M (2017). In situ coupling of Co_0.85_Se and n-doped carbon via one-step selenization of metal–organic frameworks as a trifunctional catalyst for overall water splitting and Zn–air batteries. J. Mater. Chem. A.

[35] Xu X, Song F, Hu X (2016). A nickel iron diselenide-derived efficient oxygen-evolution catalyst. Nat. Commun..

[36] Lv L, Li Z, Ruan Y, Chang Y, Ao X, Li J-G, Yang Z, Wang C (2018). Nickel–iron diselenide hollow nanoparticles with strongly hydrophilic surface for enhanced oxygen evolution reaction activity. Electrochim. Acta.

[37] Lv L, Li Z, Xue K-H, Ruan Y, Ao X (2018). Tailoring the electrocatalytic activity of bimetallic nickel–iron diselenide hollow nanochains for water oxidation. Nano Energy.

[38] Zhang J-Y, Lv L, Tian Y, Li Z, Ao X, Lan Y, Jiang J, Wang C (2017). Rational design of cobalt–iron selenides for highly efficient electrochemical water oxidation. ACS Appl. Mater. Interfaces.

[39] Hou L, Shi Y, Wu C, Zhang Y, Ma Y (2018). Monodisperse metallic NiCoSe_2_ hollow sub-microspheres: formation process, intrinsic charge-storage mechanism, and appealing pseudocapacitance as highly conductive electrode for electrochemical supercapacitors. Adv. Funct. Mater..

[40] Li J, Wan M, Li T, Zhu H, Zhao Z, Wang Z, Wu W, Du M (2018). NiCoSe_2−*x*_/*n*-doped C mushroom-like core/shell nanorods on n-doped carbon fiber for efficiently electrocatalyzed overall water splitting. Electrochim. Acta.

[41] Chen T, Tan Y (2018). Hierarchical CoNiSe_2_ nano-architecture as a high-performance electrocatalyst for water splitting. Nano Res..

[42] Zhu H, Jiang R, Chen X, Chen Y, Wang L (2017). 3D nickel–cobalt diselenide nanonetwork for highly efficient oxygen evolution. Sci. Bull..

[43] Qiu B, Cai L, Wang Y, Lin Z, Zuo Y, Wang M, Chai Y (2018). Fabrication of nickel–cobalt bimetal phosphide nanocages for enhanced oxygen evolution catalysis. Adv. Funct. Mater..

[44] Fang Z, Peng L, Qian Y, Zhang X, Xie Y, Cha JJ, Yu G (2018). Dual tuning of Ni–Co–a (*a* = P, Se, O) nanosheets by anion substitution and holey engineering for efficient hydrogen evolution. J. Am. Chem. Soc..

[45] Gong T, Qi R, Liu X, Li H, Zhang Y (2019). N, f-codoped microporous carbon nanofibers as efficient metal-free electrocatalysts for ORR. Nano-Micro Lett..

[46] Gao Z-W, Liu J-Y, Chen X-M, Zheng X-L, Mao J (2019). Engineering NiO/NiFe LDH intersection to bypass scaling relationship for oxygen evolution reaction via dynamic tridimensional adsorption of intermediates. Adv. Mater..

[47] Han X, Wu X, Zhong C, Deng Y, Zhao N, Hu W (2017). NiCo_2_S_4_ nanocrystals anchored on nitrogen-doped carbon nanotubes as a highly efficient bifunctional electrocatalyst for rechargeable zinc–air batteries. Nano Energy.

[48] Nai J, Wang S, Bai Y, Guo L (2013). Amorphous Ni(OH)_2_ nanoboxes: fast fabrication and enhanced sensing for glucose. Small.

[49] Muralee Gopi CVV, Reddy AE, Kim H-J (2018). Wearable superhigh energy density supercapacitors using a hierarchical ternary metal selenide composite of CoNiSe_2_ microspheres decorated with CoFe_2_Se_4_ nanorods. J. Mater. Chem. A.

[50] Yin J, Fan Q, Li Y, Cheng F, Zhou P, Xi P, Sun S (2016). Ni–C–N nanosheets as catalyst for hydrogen evolution reaction. J. Am. Chem. Soc..

[51] Ding Z, Tang Z, Li L, Wang K, Wu W, Chen X, Wu X, Chen S (2018). Ternary ptvco dendrites for the hydrogen evolution reaction, oxygen evolution reaction, overall water splitting and rechargeable Zn–air batteries. Inorg. Chem. Front..

[52] Gao M-R, Xu Y-F, Jiang J, Zheng Y-R, Yu S-H (2012). Water oxidation electrocatalyzed by an efficient Mn_3_O_4_/CoSe_2_ nanocomposite. J. Am. Chem. Soc..

[53] Pu Z, Luo Y, Asiri AM, Sun X (2016). Efficient electrochemical water splitting catalyzed by electrodeposited nickel diselenide nanoparticles based film. ACS Appl. Mater. Interfaces.

[54] Fang L, Li W, Guan Y, Feng Y, Zhang H, Wang S, Wang Y (2017). Tuning unique peapod-like Co(S_*x*_Se_1−*x*_)_2_ nanoparticles for efficient overall water splitting. Adv. Funct. Mater..

[55] Tang C, Cheng N, Pu Z, Xing W, Sun X (2015). NiSe nanowire film supported on nickel foam: an efficient and stable 3D bifunctional electrode for full water splitting. Angew. Chem. Int. Ed..

[56] Deng Z, Yi Q, Zhang Y, Nie H, Li G, Yu L, Zhou X (2018). Carbon paper-supported NiCo/C–N catalysts synthesized by directly pyrolyzing NiCo-doped polyaniline for oxygen reduction reaction. NANO.

[57] Timperman L, Gago AS, Alonso-Vante N (2011). Oxygen reduction reaction increased tolerance and fuel cell performance of Pt and Ru_*x*_Se_*y*_ onto oxide–carbon composites. J. Power Sour..

[58] Delacôte C, Lewera A, Pisarek M, Kulesza PJ, Zelenay P, Alonso-Vante N (2010). The effect of diluting ruthenium by iron in Ru_*x*_Se_*y*_ catalyst for oxygen reduction. Electrochim. Acta.

[59] Wang K, Tang Z, Wu W, Xi P, Liu D (2018). Nanocomposites CoPt − *x*/diatomite-C as oxygen reversible electrocatalysts for zinc-air batteries: diatomite boosted the catalytic activity and durability. Electrochim. Acta.

[60] Han X, He G, He Y, Zhang J, Zheng X (2018). Engineering catalytic active sites on cobalt oxide surface for enhanced oxygen electrocatalysis. Adv. Energy Mater..

[61] Amiinu IS, Pu Z, Liu X, Owusu KA, Monestel HGR, Boakye FO, Zhang H, Mu S (2017). Multifunctional Mo–N/C@MoS_2_ electrocatalysts for HER, OER, ORR, and Zn–air batteries. Adv. Funct. Mater..

[62] Chen Z, Wang Q, Zhang X, Lei Y, Hu W, Luo Y, Wang Y (2018). N-doped defective carbon with trace Co for efficient rechargeable liquid electrolyte-/all-solid-state Zn–air batteries. Sci. Bull..

[63] Tang C, Wang B, Wang H-F, Zhang Q (2017). Defect engineering toward atomic Co–N_*x*_–C in hierarchical graphene for rechargeable flexible solid Zn–air batteries. Adv. Mater..

[64] Yang L, Shi L, Wang D, Lv Y, Cao D (2018). Single-atom cobalt electrocatalysts for foldable solid-state Zn–air battery. Nano Energy.

[65] Guan C, Sumboja A, Zang W, Qian Y, Zhang H (2019). Decorating Co/CoNx nanoparticles in nitrogen-doped carbon nanoarrays for flexible and rechargeable zinc–air batteries. Energy Storage Mater..

[66] Li D, Zong Z, Tang Z, Liu Z, Chen S, Tian Y, Wang X (2018). Total water splitting catalyzed by Co@Ir core–shell nanoparticles encapsulated in nitrogen-doped porous carbon derived from metal–organic frameworks. ACS Sustain. Chem. Eng..

[67] Li Y, Yin J, An L, Lu M, Sun K, Zhao Y-Q, Gao D, Cheng F, Xi P (2018). FeS_2_/CoS_2_ interface nanosheets as efficient bifunctional electrocatalyst for overall water splitting. Small.

