# Supplementary material for: Hollow Nanocages of NixCo1−xSe for Efficient Zinc–Air Batteries and Overall Water Splitting
Source: Nanomicro Lett. 2019 Mar 27;11:28. doi: 10.1007/s40820-019-0258-0 (PMC7770759; doi:10.1007/s40820-019-0258-0)
Supplement: Supplementary file 1 — Supplementary material 1 (PDF 1328 kb) [file 40820_2019_258_MOESM1_ESM.pdf]

Supporting Information for

Hollow Nanocages of $Ni_xCo_{1-x}Se$ for Efficient Zinc–Air Batteries and Overall Water Splitting

Zhengxin Qian[1], Yinghuan Chen[2], Zhenghua Tang[2, 3, *], Zhen Liu[4], Xiufang Wang[1], Yong Tian[1, *], Wei Gao[1, *]

[1]School of Pharmacy, Guangdong Pharmaceutical University, Guangzhou, Guangdong 510006, People's Republic of China

[2]Guangzhou Key Laboratory for Surface Chemistry of Energy Materials, New Energy Research Institute, School of Environment and Energy, South China University of Technology, Guangzhou Higher Education Mega Center, Guangzhou, Guangdong 510006, People's Republic of China

[3]Guangdong Engineering and Technology Research Center for Surface Chemistry of Energy Materials, School of Environment and Energy, South China University of Technology, Guangzhou Higher Education Mega Centre, Guangzhou, Guangdong 510006, People's Republic of China

[4]Department of Physics & Engineering, Frostburg State University, Frostburg, MD 21532-2303, United States

*Corresponding authors. E-mail: tian_yong_tian@163.com (Yong Tian); gygaowei@126.com (Wei Gao); zhht@scut.edu.cn (Zhenghua Tang)

S1 Detailed Calculation of EASA Value

The electrochemically active surface area (EASA) of the sample was estimated from the electrochemical double-layer capacitance (C_{DL}) of the catalyst. The C_{DL} was measured via cyclic voltammograms with a potential range where no apparent Faradaic process was taking place. The double-layer charging current I_C can be related to the scan rates through Eq. S1:

$$I_C = C_{DL} \times v \qquad \text{(S1)}$$

Thus, plotting the charging currents at a specific potential against various scan rates leads to a straight line with the slope equal to C_{DL} (Fig. S7). Subsequently, the EASA value can be obtained by Eq. S2:

$$\text{EASA} = C_{DL}/C_S \qquad \text{(S2)}$$

where C_S is the capacitance measured from ideally smooth, planar surfaces of the catalyst, and here the typical value of 0.040 mF cm^{-2} for Ni is used for calculation.

S2 Detailed Calculation of Density Functional Theory

Density function theory (DFT) calculations were performed by using the CP2K package [S1]. PBE functional [S2] with Grimme D3 correction [S3] was used to describe the system. Unrestricted Kohn-Sham DFT has been used as the electronic structure method in the framework of the Gaussian and plane wave method [S4, S5]. The Goedecker-Teter-Hutter (GTH) pseudopotentials [S6, S7], DZVP-MOLOPT-GTH basis sets [S4] were utilized to describe the molecules. A plane-wave energy cut-off of 500 Ry has been employed.

The potential-dependence of reaction free energies in elementary steps involving proton-electron transfers was evaluated using the computational hydrogen electrode (CHE) approach [S8, S9]. In this approach, a reversible hydrogen electrode (RHE) is used as a reference. In alkaline medium, HER follows the Volmer-Tafel mechanism as shown in Eqs. S3-S5:

Volmer step: $H_2O + e^- \rightarrow H^* + OH^-$ (S3)

Heyrovsky step: $H_2O + e^- + H^* \rightarrow H_2 + OH^-$ (S4)

Tafel step: $H^* + H^* \rightarrow H_2$ (S5)

where * denotes the adsorption site, and denotes the hydrogen atom adsorbed on the cluster. The free energy of adsorption is estimated by assuming constant zero-point energy and entropic contributions [S10].

$\Delta G = \Delta H - \Delta TS + \Delta ZPE$ (S6)

The free energy linearly depends on the binding energy of H* as suggested in the Ref. [S9].

$\Delta G\ H^* = \Delta E\ H^* + 0.24eV$ (S7)

Where the $\Delta E\ H^*$ is the adsorption energy of H on surface.

S3 Supplementary Figures and Tables

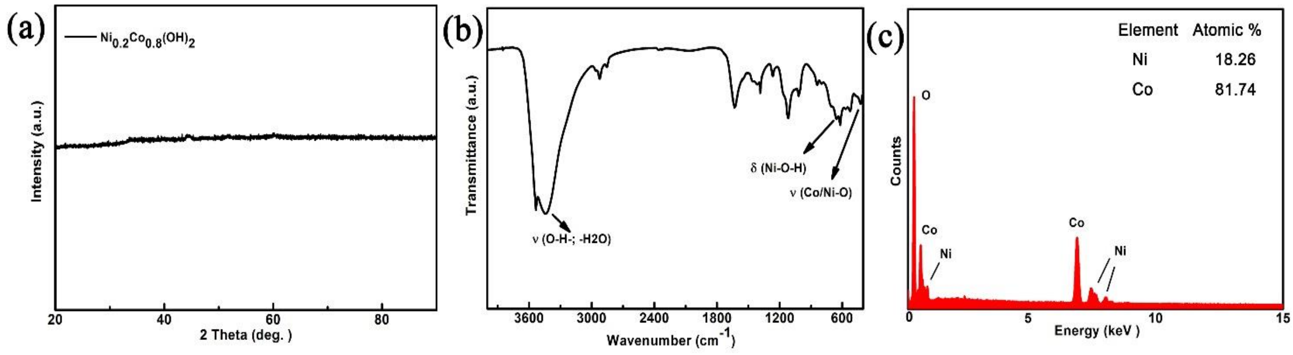

Fig. S1 a XRD pattern, **b** FT-IR spectrum, and **c** EDX spectrum of $Ni_{0.2}Co_{0.8}(OH)_2$

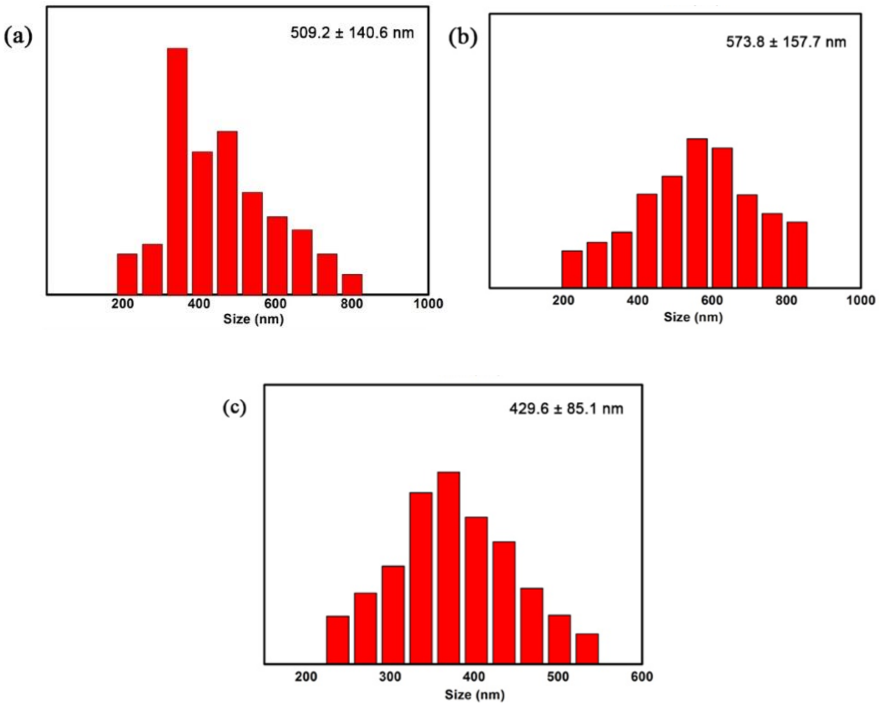

Fig. S2 Particle size distribution histograms of **a** Cu_2O, **b** $Ni_{0.2}Co_{0.8}(OH)_2$, and **c** $Ni_{0.2}Co_{0.8}Se$

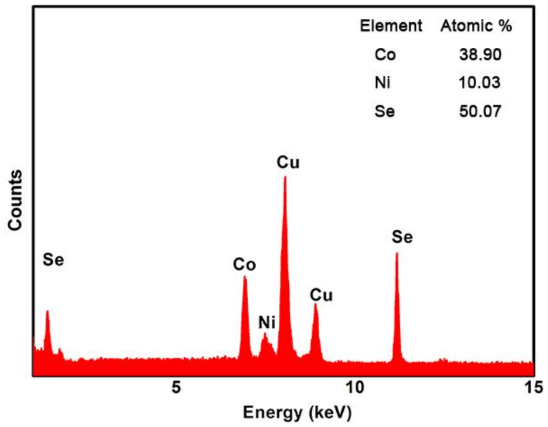

Fig. S3 EDX taken on the selected area of the $Ni_{0.2}Co_{0.8}Se$ (Cu is from the substrate)

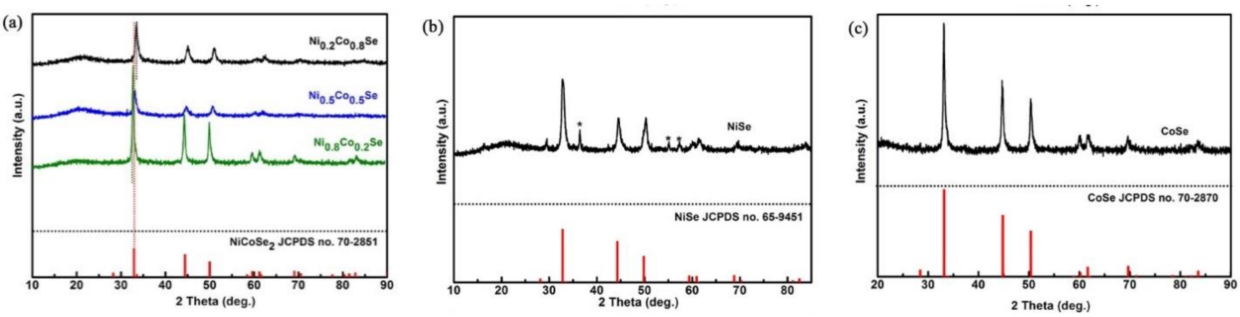

Fig. S4 XRD pattern of **a** $Ni_{0.2}Co_{0.8}Se$, $Ni_{0.5}Co_{0.5}Se$, and $Ni_{0.8}Co_{0.2}Se$; **b** NiSe. It is worth noting that the peaks deliberately marked are attributed to $NiSe_2$. **c** XRD pattern of CoSe

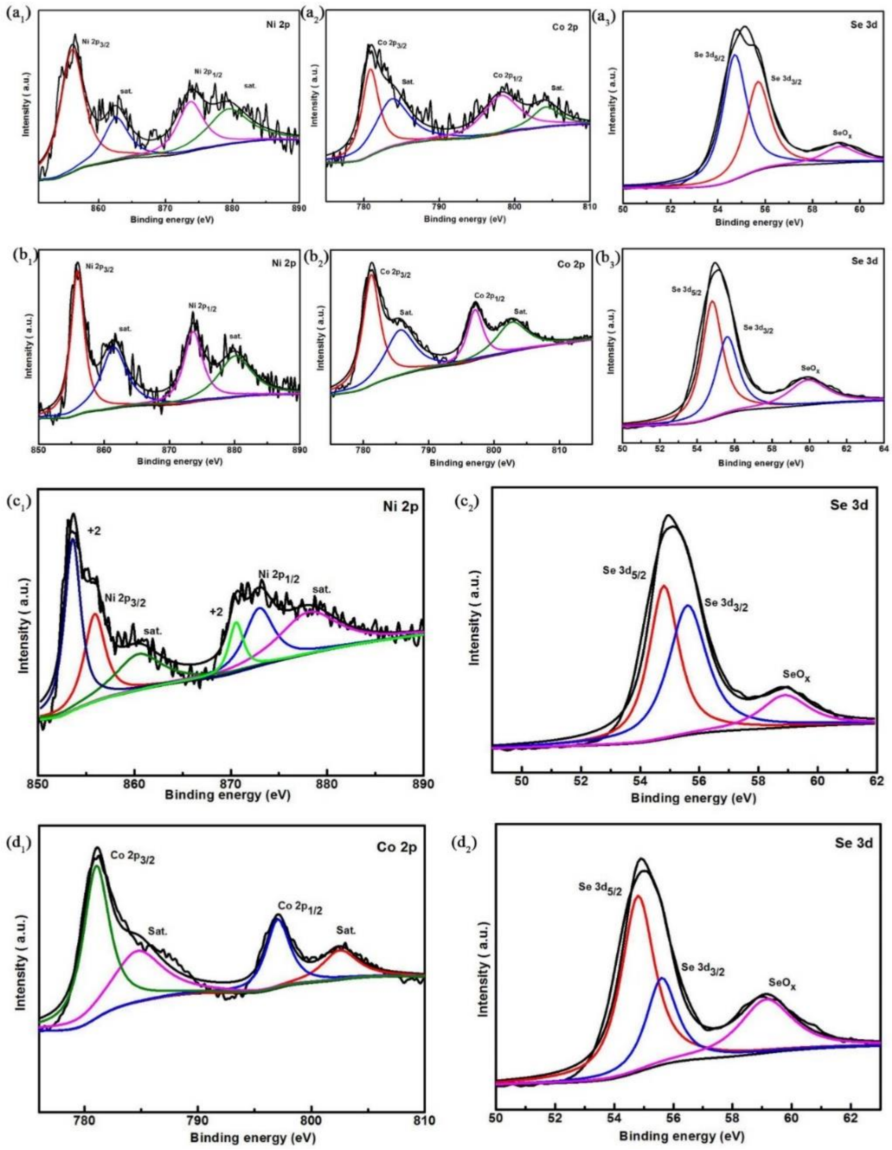

Fig. S5 The core-level XPS spectra for **a1** Ni 2p, **a2** Co 2p, and **a3** Se 3d electrons of $Ni_{0.5}Co_{0.5}Se$; **b1** Ni 2p, **b2** Co 2p, and **b3** Se 3d electrons of $Ni_{0.8}Co_{0.2}Se$; **c1** Ni 2p and **c2** Se 3d electrons of NiSe; **d1** Co 2p and **d2** Se 3d electrons of CoSe

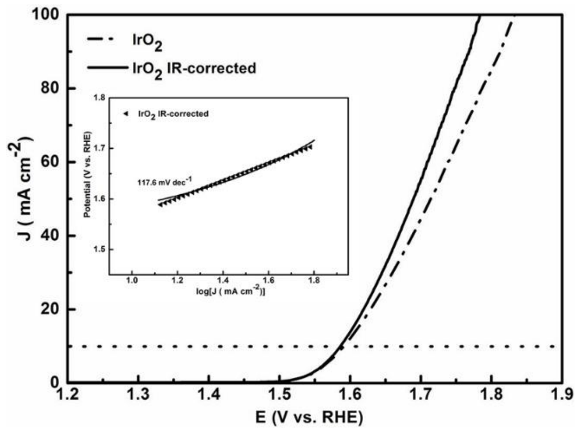

Fig. S6 OER polarization curves of IrO_2 at a scan rate of 10 mV s^{-1} in 1.0 M KOH

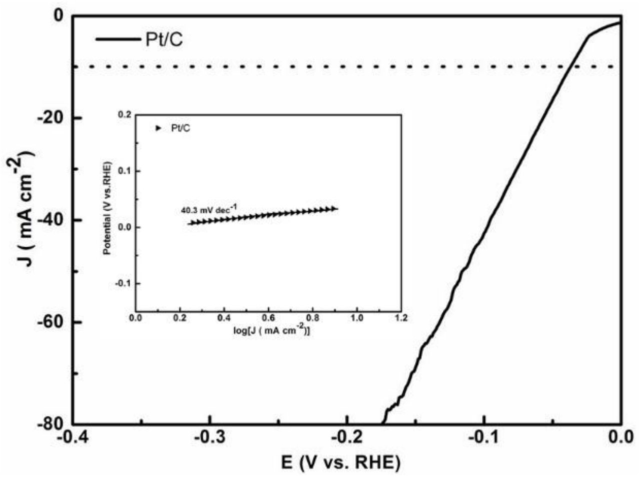

Fig. S7 HER polarization curve of Pt/C at a scan rate of 10 mV s^{-1} in 1.0 M KOH

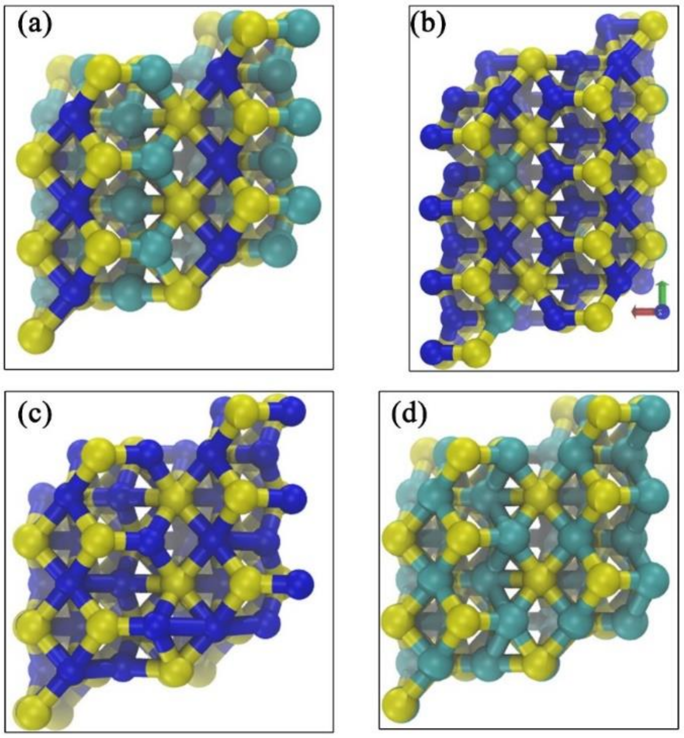

Fig. S8 Schematic diagram of **a** $Ni_{0.5}Co_{0.5}Se$, **b** $Ni_{0.8}Co_{0.2}Se$, **c** NiSe, and **d** CoSe optimal active site based on the simulation under the crystal plane (101). Color code: Yellow is Se, blue is Ni and cyan is Co

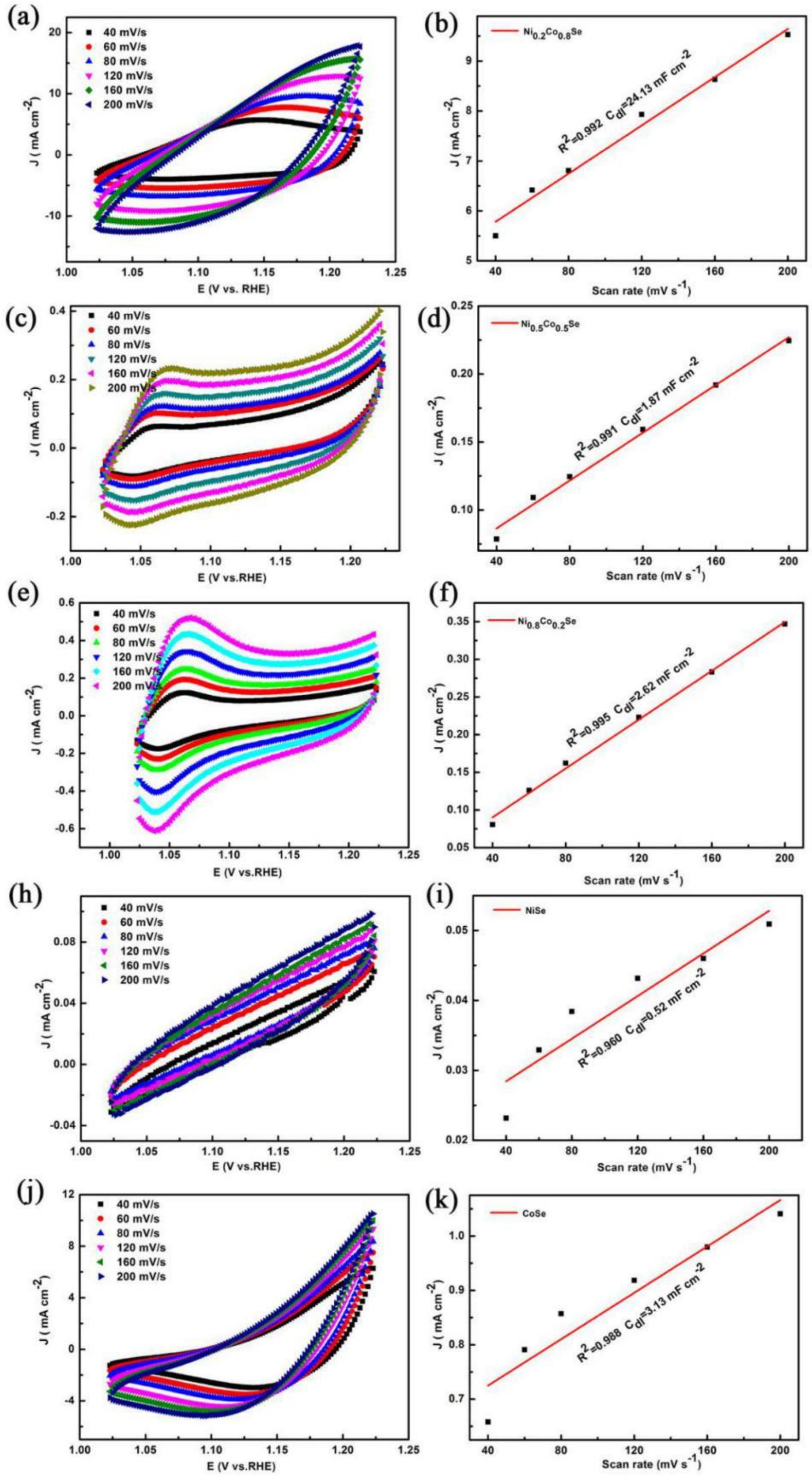

Fig. S9 Cyclic voltammogram (CV) curves of **a** $Ni_{0.2}Co_{0.8}Se$, **c** $Ni_{0.5}Co_{0.5}Se$, **e** $Ni_{0.8}Co_{0.2}Se$, **h** NiSe, **j** CoSe modified electrodes in the double layer region at scan rates of 40, 60, 80, 120, 160, and 200 mV s^{-1} in 1.0 M KOH. Their corresponding current density plot (**b-k**) as a function of scan rate derived from (**a-j**), respectively.

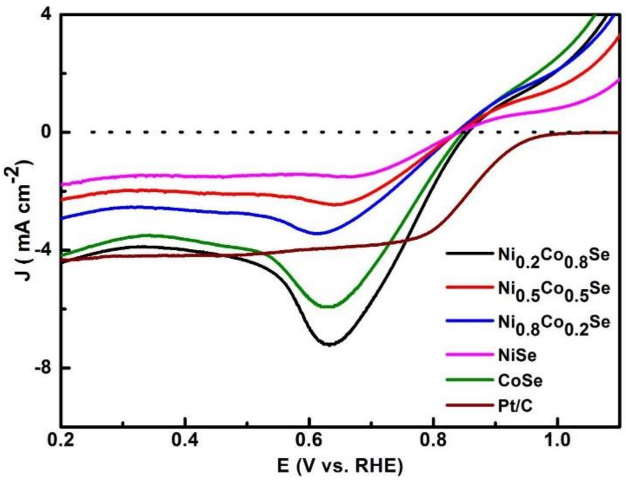

Fig. S10 The LSV curves of the $Ni_xCo_{1-x}Se$ series and Pt/C for ORR in 0.1 M KOH

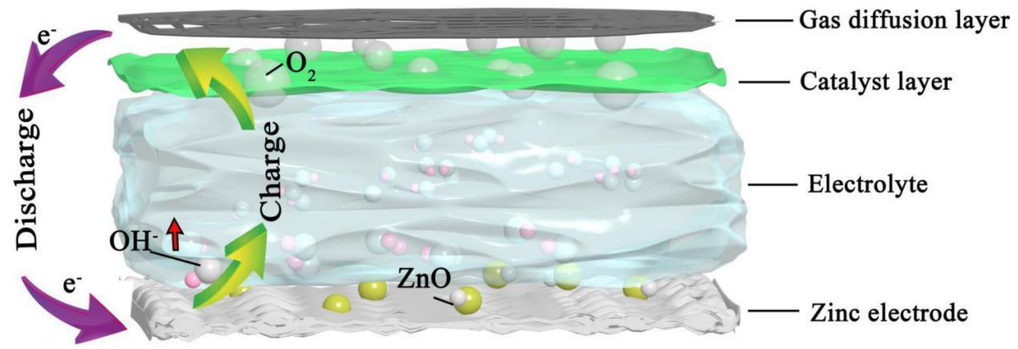

Fig. S11 Schematic representation of a Zn−air battery

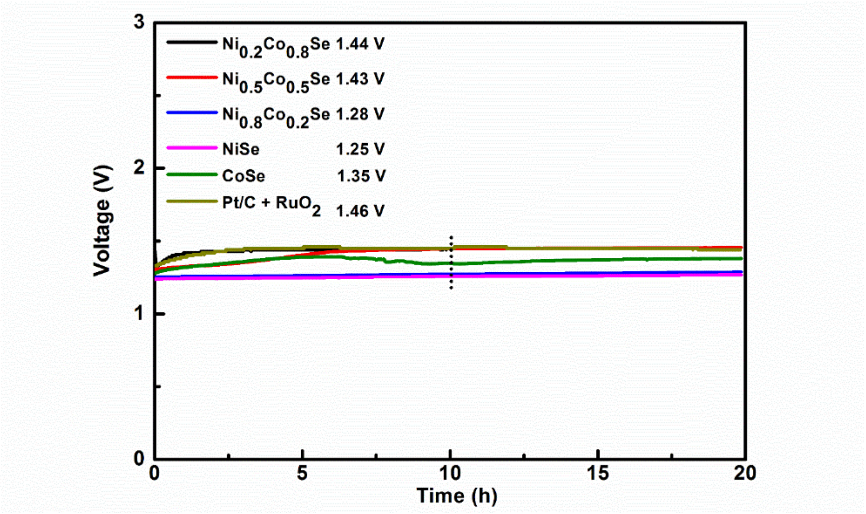

Fig. S12 Open-circuit potential sketch of $Ni_xCo_{1-x}Se$ and commercial Pt/C+RuO_2 catalyst. The value was taken at about 10 h

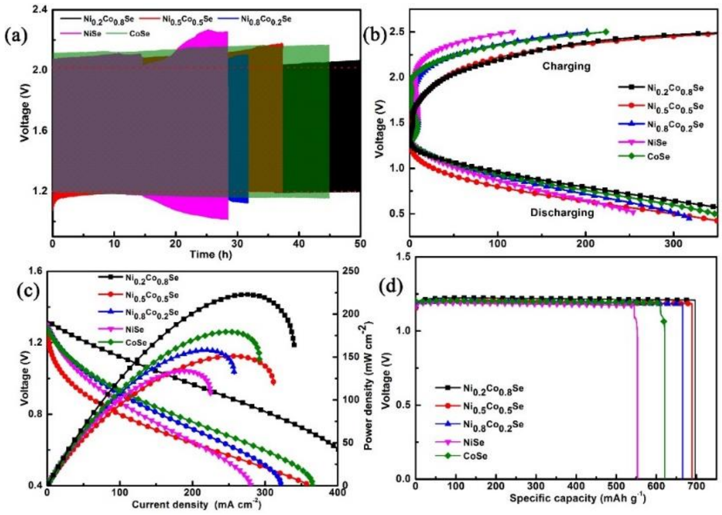

Fig. S13 a Cycling performance of the rechargeable ZAB using a series of $Ni_xCo_{1-x}Se$ at 10 mA cm^{-2} with each cycle of 5 min. **b** Charge and discharge polarization curves of the rechargeable ZAB using a series of $Ni_xCo_{1-x}Se$. **c** Polarization and power density curves of the $Ni_xCo_{1-x}Se$. **d** The specific capacities of $Ni_xCo_{1-x}Se$ at 10 mA cm^{-2}

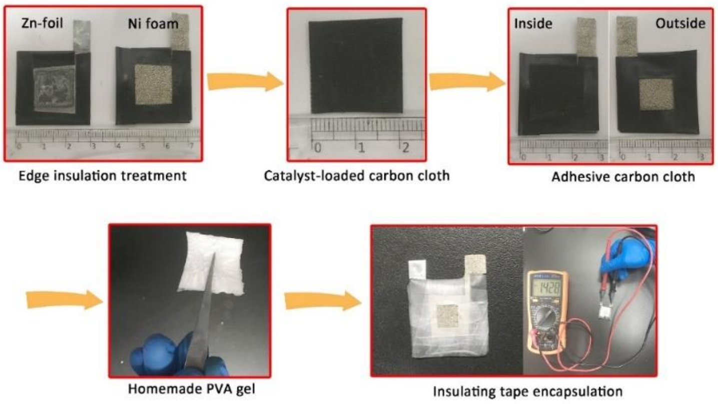

Fig. S14 Schematic diagram of the preparation process of an all-solid-state ZAB

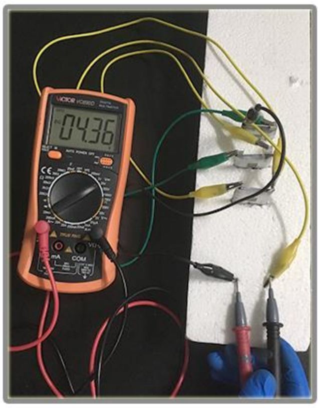

Fig. S15 Open circuit voltmeter diagram of three all-solid-state zinc-air batteries prepared from the $Ni_{0.2}Co_{0.8}Se$ catalyst

Table S1 Summary of OER and HER catalytic activities of the selenide and relevant leading catalysts reported in previous work

Catalyst	OER			HER			References
	Electrolyte	$E_{10,\ overpotential}$ (mV vs. RHE)	Tafel slope (mV dec^{-1})	Electrolyte	$E_{10,\ overpotential}$ (mV vs. RHE)	Tafel slope (mV dec^{-1})	
**$Ni_{0.2}Co_{0.8}Se$**	1 M KOH	280	86.8	1 M KOH	73	54.8	This work
**$NiSe_2$/Ti**	1 M KOH	295	82	1 M KOH	70	82	[53]
**$CoSe_2$/ Mn_3O_4**	1 M KOH	450	49	/	/	/	[52]
**NiSe/NF**	1 M KOH	270	64	1 M KOH	96	120	[55]
**Ni_xSe ($0.5 \leqslant x \leqslant 1$)**	1 M KOH	330	51	1 M KOH	233	86	[33]
**$Co_{0.85}Se$**	1 M KOH	320	75	1 M KOH	230	125	[34]
**$Ni_{0.75}Fe_{0.25}Se_2$**	1 M KOH	272	73	/	/	/	[36]
**$Co(S_{0.22}Se_{0.78})_2$**	1 M KOH	283	65.5	1 M KOH	175	117.9	[54]

Table S2 The performance comparison of $Ni_{0.2}Co_{0.8}Se$ and recently documented similar materials in the application of liquid zinc-air battery and all solid-state zinc-air battery

Catalyst	Liquid zinc-air battery		All-solid-state zinc-air battery			References
	Open circuit voltage (V)	Power density (mW cm^{-2})	Open circuit voltage (V)	Round trip efficiency (%)/ Cycling condition (mA cm^{-2})	Power density (mW cm^{-2})	
**$Ni_{0.2}Co_{0.8}Se$**	1.44	223.5	1.428	61.96/ 2	41.03	This work
**CoN_4/NG**	1.51	115.0	/	~60.45/ 1	28	[64]
**NGM-Co**	1.439	152.0	1.439	63.00/1	29	[63]
**NC-Co/CoN_x**	/	/	1.40	/1	41.5	[65]
**Co-NDC**	1.30	154.0	~1.25	/2	45.9	[62]
**Co_3O_4/N-rGO**	/	/	1.314	~60/3	36.1	[29]

Supplementary References

[S1] J. Hutter, M. Iannuzzi, F. Schiffmann, J. VandeVondele, Cp2k: Atomistic simulations of condensed matter systems. WIREs Comput. Mol. Sci. **4**(1), 15-25 (2014). https://doi.org/doi:10.1002/wcms.1159

[S2] J.P. Perdew, K. Burke, M. Ernzerhof, Generalized gradient approximation made simple. Phys. Rev. Lett. **77**(18), 3865-3868 (1996). https://doi.org/10.1103/PhysRevLett.77.3865

[S3] S. Grimme, Semiempirical gga-type density functional constructed with a long-range dispersion correction. J. Comput. Chem. **27**(15), 1787-1799 (2006). https://doi.org/doi:10.1002/jcc.20495

[S4] J. VandeVondele, J. Hutter, Gaussian basis sets for accurate calculations on molecular systems in gas and condensed phases. J. Chem. Phys. **127**(11), 114105 (2007). https://doi.org/10.1063/1.2770708

[S5] J. VandeVondele, M. Krack, F. Mohamed, M. Parrinello, T. Chassaing, J. Hutter, Quickstep: Fast and accurate density functional calculations using a mixed gaussian and plane waves approach. Comp. Phys. Comm. **167**(2), 103-128 (2005). https://doi.org/https://doi.org/10.1016/j.cpc.2004.12.014

[S6] S. Goedecker, M. Teter, J. Hutter, Separable dual-space gaussian pseudopotentials. Phys. Rev. B **54**(3), 1703-1710 (1996). https://doi.org/10.1103/PhysRevB.54.1703

[S7] C. Hartwigsen, S. Goedecker, J. Hutter, Relativistic separable dual-space gaussian pseudopotentials from H to Rn. Phys. Rev. B **58**(7), 3641-3662 (1998). https://doi.org/10.1103/PhysRevB.58.3641

[S8] J.K. Nørskov, J. Rossmeisl, A. Logadottir, L. Lindqvist, J.R. Kitchin, T. Bligaard, H. Jónsson, Origin of the overpotential for oxygen reduction at a fuel-cell cathode. J. Phy. Chem. B **108**(46), 17886-17892 (2004). https://doi.org/10.1021/jp047349j

[S9] J.K. Nørskov, T. Bligaard, A. Logadottir, J.R. Kitchin, J.G. Chen, S. Pandelov, U. Stimming, Trends in the exchange current for hydrogen evolution. J. Elec. Soc. **152**(3), J23-J26 (2005). https://doi.org/10.1149/1.1856988

[S10] B. Hinnemann, P.G. Moses, J. Bonde, K.P. Jørgensen, J.H. Nielsen, S. Horch, I. Chorkendorff, J.K. Nørskov, Biomimetic hydrogen evolution: MoS_2 nanoparticles as catalyst for hydrogen evolution. J. Am. Chem. Soc. **127**(15), 5308-5309 (2005). https://doi.org/10.1021/ja0504690
